# HDAC1 modulates OGG1-initiated oxidative DNA damage repair in the aging brain and Alzheimer’s disease

**DOI:** 10.1038/s41467-020-16361-y

**Published:** 2020-05-18

**Authors:** Ping-Chieh Pao, Debasis Patnaik, L. Ashley Watson, Fan Gao, Ling Pan, Jun Wang, Chinnakkaruppan Adaikkan, Jay Penney, Hugh P. Cam, Wen-Chin Huang, Lorena Pantano, Audrey Lee, Alexi Nott, Trongha X. Phan, Elizabeta Gjoneska, Sara Elmsaouri, Stephen J. Haggarty, Li-Huei Tsai

**Affiliations:** 1 Picower Institute for Learning and Memory, Massachusetts Institute of Technology, Cambridge, MA 02139 USA; 2 Department of Brain and Cognitive Sciences, Massachusetts Institute of Technology, Cambridge, MA 02139 USA; 3 Chemical Neurobiology Laboratory, Departments of Neurology and Psychiatry, Massachusetts General Hospital and Harvard Medical School, Boston, MA 02114 USA; 4 Present Address: Caltech Bioinformatics Resource Center at Beckman Institute of Caltech, Pasadena, CA 91225 USA; 5 Present Address: Department of Cellular and Molecular Medicine, University of California, San Diego, La Jolla, CA 92093 USA; 6 Present Address: Neurobiology Laboratory, National Institute of Environmental Health Sciences, National Institutes of Health, Research Triangle Park, NC 27709 USA

**Keywords:** DNA damage and repair, Cognitive ageing, Alzheimer's disease, Neurodegeneration, Learning and memory

## Abstract

DNA damage contributes to brain aging and neurodegenerative diseases. However, the factors stimulating DNA repair to stave off functional decline remain obscure. We show that HDAC1 modulates OGG1-initated 8-oxoguanine (8-oxoG) repair in the brain. HDAC1-deficient mice display age-associated DNA damage accumulation and cognitive impairment. HDAC1 stimulates OGG1, a DNA glycosylase known to remove 8-oxoG lesions that are associated with transcriptional repression. HDAC1 deficiency causes impaired OGG1 activity, 8-oxoG accumulation at the promoters of genes critical for brain function, and transcriptional repression. Moreover, we observe elevated 8-oxoG along with reduced HDAC1 activity and downregulation of a similar gene set in the 5XFAD mouse model of Alzheimer’s disease. Notably, pharmacological activation of HDAC1 alleviates the deleterious effects of 8-oxoG in aged wild-type and 5XFAD mice. Our work uncovers important roles for HDAC1 in 8-oxoG repair and highlights the therapeutic potential of HDAC1 activation to counter functional decline in brain aging and neurodegeneration.

## Abstract (web summary)

Defects in DNA repair have been linked to brain aging and neurodegenerative disorders. Here the authors reveal a role for HDAC1 in stimulating OGG1 activity to alleviate 8-oxoG lesions with implications in the aging brain and neurodegenerative diseases.

## Introduction

Progressive deterioration of cellular and physiological functions is inevitable symptoms of aging, increasing vulnerability to diseases, and mortality^1^. Genomic instability due to accumulation of DNA damage is a hallmark of aging^2^, and has been linked to DNA mutations, altered gene expression, and cognitive impairment in the human brain^3,4^. Consistently, mutations in DNA repair genes and impaired DNA repair pathways are associated with premature aging and neurological symptoms in humans and rodents^5–7^, while upregulation of DNA repair pathways is found in long-lived rodents and reducing DNA damage in model systems improves outcomes^8–10^. A better understanding of the mechanisms regulating DNA damage and repair in the brain holds potential for identifying interventions to combat aging and disease.

Impaired DNA damage repair pathways are associated with numerous neurodegenerative conditions^7^. In Alzheimer’s disease (AD), post-mortem patient brains exhibit elevated DNA double-strand breaks (DSBs) and reduced expression of DSB repair factors^11,12^, and AD brain extracts show reduced DSB repair capacity in vitro^13^, reflecting impaired non-homologous end joining (NHEJ). In mouse models such as CK-p25 and Tau P301S, elevated DNA damage is observed at pre-symptomatic stages before the appearance of neurological symptoms or neurodegeneration^14,15^. Haploinsufficiency for DNA polymerase β, an enzyme involved in base excision DNA repair, exacerbates pathologies in 3xTg mice^16^. Thus, elevated DNA damage likely predisposes for neurodegenerative disease, and enhancing DNA repair may forestall functional decline in brain aging and neurodegeneration^9,10^.

Histone deacetylases (HDACs) are enzymes that remove acetyl groups from lysine residues of histones and non-histone proteins. HDACs modulate many cellular processes, including transcription, chromatin remodeling, and DNA repair^17^. We have previously uncovered functions for HDAC1, a class I HDAC, in maintaining genomic integrity in cultured neurons and the mouse brain^14,15,18^. DSB triggers HDAC1 recruitment to break sites to deacetylate H3 at lysine 56 (H3K56) and histone H4 at lysine 16 (H4K16), which promotes DSB repair through NHEJ^19^, the major DSB repair pathway in neurons^7^. However, it remains unclear whether HDAC1 plays a role in other DNA repair pathways, and whether HDAC1 is required for proper gene expression and normal brain functions during aging.

Here, we examine the effects of conditional *Hdac1* deletion (*Hdac1* cKO) in neurons and astrocytes on brain function. Despite the lack of gross brain abnormalities, *Hdac1* cKO mice exhibited age-dependent cognitive decline. Although HDAC1 is a well-known transcriptional repressor^17^, a majority of differentially expressed genes (DEGs) in aged *Hdac1* cKO mice were downregulated. Intriguingly, the promoters of many downregulated genes contain a guanine-rich sequence known to be susceptible to oxidative DNA damage, which became enriched with 8-oxoguanine (8-oxoG) DNA lesions in aged brains. We show that HDAC1 interacts with and deacetylates 8-oxoGDNA glycosylase 1 (OGG1), a DNA glycosylase responsible for 8-oxoG lesion removal^20^, enhancing its cleavage activity. *Hdac1* ablation resulted in reduced OGG1 activity, coinciding with elevated 8-oxoG lesions at the promoters of susceptible genes and their transcriptional repression. We observe increased 8-oxoG lesions in 5XFAD mice, an AD model^21^, and a significant overlap with genes downregulated in aged *Hdac1* cKO mice. We demonstrate that HDAC1 activity is impaired in 5XFAD mice, and that HDAC1 deficiency in 5XFAD mice exacerbates 8-oxoG lesions. Furthermore, pharmacological activation of HDAC1 stimulated OGG1 activity, reduces 8-oxoG lesions in aged wild-type and 5XFAD mice, and alleviates 5XFAD cognitive deficits. Our findings uncover a role for HDAC1 in modulating DNA repair of 8-oxoG lesions and highlight the therapeutic potential of pharmacological HDAC1 activation in brain aging and neurodegeneration.

## Results

### Aged *Hdac1* cKO mice display astrogliosis and DNA damage

To understand HDAC1 functions in the brain, and to circumvent embryonic lethality associated with germline deletion of *Hdac1*^22^, we generated *Hdac1* brain-specific cKO mice by crossing *Hdac1*^*f/f*^ mice^23^ to those expressing *Nestin-Cre*^24^, resulting in *Hdac1* deletion in neurons and astrocytes. Indeed, we observed loss of HDAC1 in hippocampal lysates of 3-month-old *Hdac1* cKO animals (Supplementary Fig. 1a). Immunohistochemistry confirmed HDAC1 absent in neurons and astrocytes, but detectable in microglia (Supplementary Fig. 1b).

**Fig. 1:**
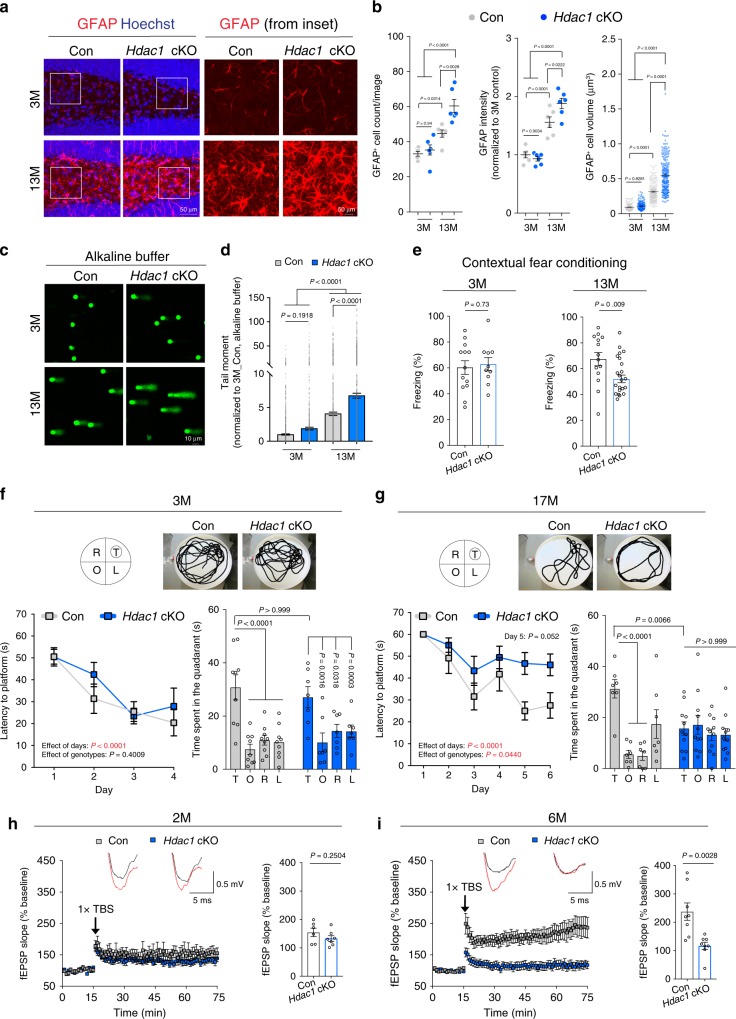
*Hdac1* cKO mice exhibit age-associated impairments. **a** Representative images of control and *Hdac1* cKO mice at the age of 3 months (3M) and 13 months (13M) stained for GFAP (red) and nuclei Hoechst (blue) in the hippocampal region. **b** Analysis of GFAP^+^ cells. **a**, **b** Three mice were analyzed in each group (two sections/mouse). Cells analyzed for cell volume were: 144 (3M control), 168 (3M *Hdac1* cKO), 181 (13M control), and 227 (13M *Hdac1* cKO). **c**, **d** Representative images and quantification of the comet assay using hippocampal homogenates. **c**, **d** Nuclei analyzed (three mice/group) were: 1356 (3M control), 1268 (3M *Hdac1* cKO), 1898 (13M control), and 1944 (13M *Hdac1* cKO). **e** Analysis of freezing behavior during contextual fear conditioning test. Animals analyzed were: 13 (3M control), 10 (3M *Hdac1* cKO), 14 (13M control), and 22 (13M *Hdac1* cKO). **f**, **g** Control and *Hdac1* cKO mice were subjected to the Morris water maze (MWM) test. Shown are the latencies to hidden platform during training and time spent in each quadrant during the probe trial. Images show the location of each quadrant and swim trace of mouse during the probe trial. T = target, O = opposite, R = right, L = left. Animals analyzed were: 9 (3M control), 7 (3M *Hdac1* cKO), 7 (17M control), and 11 (17M *Hdac1* cKO). **h**, **i** Hippocampal LTP induction in control and *Hdac1* cKO mice. Animals/slices analyzed were: 3/6 (2M control), 3/7 (2M *Hdac1* cKO), 4/8 (6M control), and 5/8 (6M *Hdac1* cKO). Overlay of the representative field excitatory post-synaptic potential slope (fEPSPs) before (black trace) and after (red trace) LTP induction. Theta-burst stimulation (TBS). All values are shown as mean ± SEM. Statistical analysis: **b**, **d** one-way ANOVA with Tukey’s post hoc test; **e**, **h**, **i** two-tailed Student’s *t* test; **f**, **g** two-way repeated-measures ANOVA with Bonferroni’s post hoc test for acquisition phase; two-way ANOVA with Bonferroni’s post hoc test for probe trial. Source data are provided as a Source Data file.

Young (3-month-old) cKO animals displayed no gross abnormalities in brain organization. Immunohistochemistry showed no significant changes in cellular abundance or distribution of neurons, astrocytes, and microglia between young *Hdac1* cKO mice and age-matched *Hdac1*^*f/f*^ controls (Supplementary Fig. 1c, d). We next examined aged (13-month-old) animals, and found that whereas the number of neurons and microglia were similar in the aged hippocampus (Supplementary Fig. 1c, d), there was a significant increase in astrocyte number and glial fibrillary acidic protein (GFAP) immunoreactivity (Fig. 1a, b), indicative of astrogliosis. HDAC1 deficiency did not alter the number of apoptotic cells in the mouse brain (Supplementary Fig. 1e, f). Astrocytes displayed age-dependent increase in size in aged versus young controls (Fig. 1a, b), as quantified by 3D volume rendering of GFAP signals, consistent with their reported morphological changes and hypertrophic processes as the brain ages^25^. Notably, astrocytic hypertrophy and process ramification were enhanced in aged *Hdac1* cKO mice (Fig. 1a, b). No difference was observed in GFAP intensity, astrocyte number, or size in young *Hdac1* cKO versus control mice (Fig. 1a, b). Thus, HDAC1 deficiency results in astrogliosis, increased GFAP immunoreactivity, and astrocytic hypertrophy.

We previously reported that cultured neurons lacking HDAC1 exhibit increased DNA damage^14,15^. To assess the effect of HDAC1 deficiency on DNA damage in vivo, we performed the comet assay on hippocampal homogenates of *Hdac1* cKO and control mice. Single-cell suspensions are embedded in agarose before subjecting to electrophoresis. As damaged DNA migrates faster, nuclei with DNA damage exhibit a comet-like morphology, with longer length or higher intensity of the DNA in the comet “tail” indicating increased DNA damage and quantified as “tail moment” (tail length × tail DNA%)^26^. Electrophoresis, when performed in an alkaline buffer, enables detection of multiple classes of DNA damage^26^. We observed no difference in tail moment when comparing young *Hdac1* cKO mice to controls; however, tail moment was increased in aged versus young controls (Fig. 1c, d), indicating increased DNA damage in the aged brain. Importantly, HDAC1 loss significantly elevated tail moment compared to controls in aged mice (Fig. 1c, d). These findings support important roles for HDAC1 in maintaining genomic integrity during brain aging.

### *Hdac1* cKO mice exhibit age-dependent cognitive decline

We next assessed whether HDAC1 deficiency impacts cognitive function. We first conducted contextual fear conditioning to evaluate hippocampal-dependent spatial memory^27^. Contextual memory, as measured by freezing behavior, was unaltered in young *Hdac1* cKO mice (Fig. 1e). Notably, aged *Hdac1* cKO mice exhibited a 22.2% reduction in freezing behavior compared to controls. Locomotor activity, which can affect freezing behavior^27^, was unaltered in aged *Hdac1* cKO mice (Supplementary Fig. 1g). We also subjected *Hdac1* cKO mice to the Morris water maze task that assesses hippocampal-dependent spatial learning^28^. HDAC1 deficiency did not alter cognitive performance in young animals, while aged cKO mice showed increased escape latency compared to controls (Fig. 1f, g). Moreover, these mice exhibited impaired memory recall, as demonstrated by a 50.3% reduction in time spent in the target quadrant compared to controls during the probe trial (Fig. 1g). Cognitive deficits observed in aged *Hdac1* cKO mice were not due to a change in swimming velocity nor impaired vision (Supplementary Fig. 1h, i). We next tested whether HDAC1 deficiency could impair long-term potentiation (LTP), potentially underlying their memory dysfunction in aging^29^. While hippocampal LTP induction was not altered at 2 months of age, we could already detect impairments in 6-month-old *Hdac1* cKO mice (Fig. 1h, i). Thus, *Hdac1* cKO mice exhibit age-dependent deficits in memory function and LTP induction, concomitant with increased DNA damage.

### HDAC1 controls a unique gene expression program in aging

HDACs are known to repress gene expression by collaborating with transcriptional co-repressors at gene promoters^30^. We performed differential gene expression analysis via RNA-sequencing (RNA-seq) to determine whether altered gene expression in *Hdac1* cKO mice contributes to the aforementioned phenotypes. RNA-seq analysis of hippocampal tissues from young mice revealed 115 DEGs (52 upregulated genes, 63 downregulated genes, *Hdac1* cKO versus controls; Supplementary Fig. 2a). To understand how HDAC1 regulates these genes, we performed HDAC1 chromatin immunoprecipitation (ChIP) followed by sequencing (ChIP-seq) using hippocampal tissues from young wild-type animals. We identified 7014 HDAC1 binding sites enriched at promoters and 5′-untranslated regions (Supplementary Fig. 2b, c). Intersecting RNA-seq and ChIP-seq datasets showed that only 24.3% of DEGs (7 upregulated and 21 downregulated genes) have HDAC1 binding at their promoters (Supplementary Fig. 2a). The expression of the majority of genes with HDAC1 binding in wild-type mice was not altered in young *Hdac1* cKO animals, indicating that HDAC1 may not directly regulate their expression under normal circumstances.

We next performed RNA-seq on hippocampal tissues from aged *Hdac1* cKO mice and controls and observed widespread gene expression differences in aged animals, with 479 DEGs identified between aged *Hdac1* cKO mice and controls, several of which were validated by quantitative PCR (qPCR) analysis (Supplementary Fig. 2d, e). Surprisingly, despite the established roles for HDACs as transcriptional repressors^17^, most DEGs (397 genes, 82.8%) were downregulated in aged *Hdac1* cKO mice (Supplementary Fig. 2d), and of these most (77.8%) had no detectable HDAC1 binding at their promoters (Supplementary Fig. 2d). These findings indicate that loss of HDAC1 alters gene expression in an age-dependent manner, likely via non-canonical mechanisms independent of direct promoter binding.

**Fig. 2:**
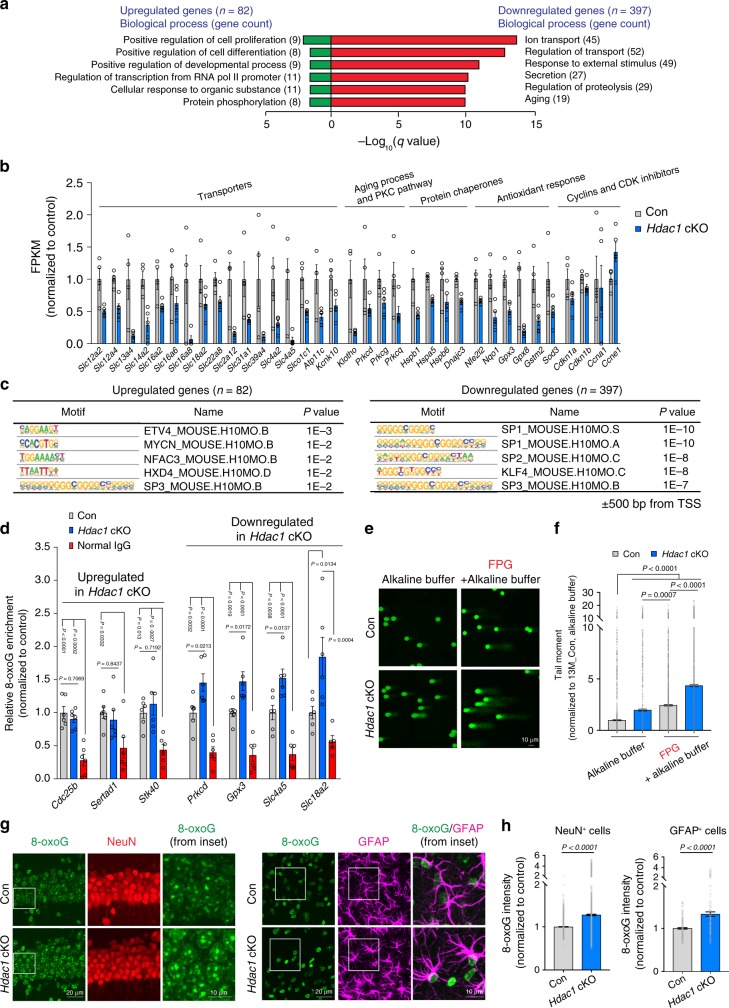
Promoters of downregulated genes in aged *Hdac1* cKO mice are enriched for guanine-rich motifs and 8-oxoG lesions. **a** Enriched GO terms of DEGs from 13M animals were annotated using MSigDB^31,32^. **b** FPKM (Fragments Per Kilobase of transcript per Million mapped reads) values of DEGs identified in 13M *Hdac1* cKO mice function in ion transport, aging process, PKC pathway, protein chaperone, and antioxidant response. Cell cycle-related genes were unaltered. **c** Known motifs identified by HOMER program^40^ within upstream and downstream (±) 500 base pairs (bp) from TSS (transcriptional start site) of DEGs. **d** ChIP-qPCR analysis indicates the enrichment of 8-oxoG at gene promoters (500 bp upstream of TSS). At least five animals were analyzed from two independent experiments. **e**, **f** Representative images and quantification plots of the alkaline and FPG-modified comet assay using hippocampal homogenates from 13M control and *Hdac1* cKO mice. **e**, **f** Nuclei analyzed (three mice/group) were: alkaline, 1928 (13M control), 1412 (13M *Hdac1* cKO); FPG + alkaline, 1872 (13M control) and 1500 (13M *Hdac1* cKO). **g** Representative images of 13M control and *Hdac1* cKO mice stained for 8-oxoG (green), NeuN (red), and GFAP (magenta). **h** Quantification plots showing the 8-oxoG intensity. **g**, **h** Four mice were analyzed (two sections/mouse). Cells analyzed were: NeuN^+^ cells, 1585 (13M control), 2306 (13M *Hdac1* cKO); GFAP^+^ cells, 191 (13M control), 164 (13M *Hdac1* cKO). All values are shown as mean ± SEM. Statistical analysis: **a** weighted Kolmogorov–Smirnov-like test followed by multiple hypothesis testing; **c** cumulative binomial distributions; **d**, **f** one-way ANOVA with Tukey’s post hoc test; **h** two-tailed Student’s *t* test. Source data are provided as a Source Data file.

We used gene ontology (GO) analysis^31,32^ to examine cellular pathways and processes affected by HDAC1 deficiency in the aged brain and identified upregulated genes associated with generalized cellular functions. In contrast, downregulated genes were more specific to brain function and aging, having GO terms related to ion transport, response to external stimulus, proteolysis, and aging (Fig. 2a). For instance, ion transport includes members of solute carrier transporter family important for neuronal membrane polarization and synaptic transmission^33^, and *Klotho* plus several isoforms of protein kinase C (PKC) were also downregulated in aged *Hdac1* cKO mice (Fig. 2b). *Klotho* is a known anti-aging gene whose overexpression extends lifespan in mice^34^, and whose loss results in shortened lifespan, premature aging, and cognitive deficits^35,36^. The PKC pathway is important for learning and memory^37^, and reduced levels of multiple PKC isoforms have been observed in the aged human cortex^4^. Downregulated genes in aged *Hdac1* cKO mice were also enriched for protein chaperones and factors involved in antioxidant responses (Fig. 2b), two pathways that have been implicated in brain aging and neurodegenerative disorders^38,39^, but were not enriched in young *Hdac1* cKO mice (Supplementary Fig. 2f). Together, HDAC1 deficiency in aged mouse brains dampened the expression of genes important for brain function.

### 8-oxoG accumulation and gene repression due to *Hdac1* cKO

To gain insight into how gene expression observed in aged *Hdac1* cKO mice may be regulated, we analyzed gene promoters for enrichment of transcription factor (TF) binding sites^40^. The most enriched motifs among downregulated genes were the consensus binding sites for the specificity protein (SP) and Krüppel-like factor (KLF) (SP/KLF) family of TFs, which are composed of stretches of guanine-rich sequences (Fig. 2c). Guanine bases are susceptible to reactive oxygen species (ROS)-induced 8-oxoG DNA lesions^41^, known to accumulate in the aging brain^42^. Unrepaired 8-oxoG lesions can interfere with TF binding and disrupt gene expression^43,44^, as has been observed in the aging human cortex and in peripheral tissues of Fanconi anemia patients^4,45^. Accordingly, we examined 8-oxoG accumulation in *Hdac1* cKO mice.

We first determined whether the promoters of genes downregulated in aged *Hdac1* cKO mice were burdened with increased 8-oxoG lesions. We performed ChIP using an 8-oxoG antibody followed by qPCR to probe the promoters of seven representative DEGs chosen based on their promoters harboring SP/KLF family binding sites and comparable percentage of guanine bases (500 base pairs (bp) upstream from transcription start site). These included three upregulated genes (*Cdc25b*, *Sertad1*, and *Stk40*) and four downregulated genes (*Prkcd*, *Gpx3*, *Slc4a5*, and *Slc18a2*). *Hdac1* ablation led to elevated 8-oxoG at promoters of all four downregulated genes in aged mice (Fig. 2d). In contrast, no such enrichment was detected for the three upregulated genes. These findings suggest that 8-oxoG accumulation at promoters of downregulated genes is likely linked to their transcriptional repression in aged *Hdac1* cKO mice.

To determine whether 8-oxoG lesions increase globally in aged *Hdac1* cKO mice, we performed a modified comet assay in which hippocampal homogenates were first incubated with formamidopyrimidine DNA glycosylase (FPG), an enzyme that removes 8-oxoG to generate single-strand breaks, enabling the detection of oxidative DNA lesions^46^. We found that aged *Hdac1* cKO mice exhibited higher tail moment compared to controls (Fig. 2e, f), indicative of increased 8-oxoG lesions. To assess which cell types exhibit increased 8-oxoG levels in aged *Hdac1* cKO mice, we co-stained brain slices with 8-oxoG and cell-type makers (NeuN for neurons; GFAP for astrocytes). In aged *Hdac1* cKO mice, elevated 8-oxoG immunoreactivity was observed in both neurons and astrocytes, indicating that HDAC1 maintains genomic integrity in glia as well as neurons (Fig. 2g, h). These findings link HDAC1 deficiency to elevated oxidative DNA lesions, likely attenuating expression for a subset of aging-related genes with guanine-rich motifs in their promoters.

### HDAC1 deacetylates OGG1 to stimulate its cleavage activity

**Fig. 3:**
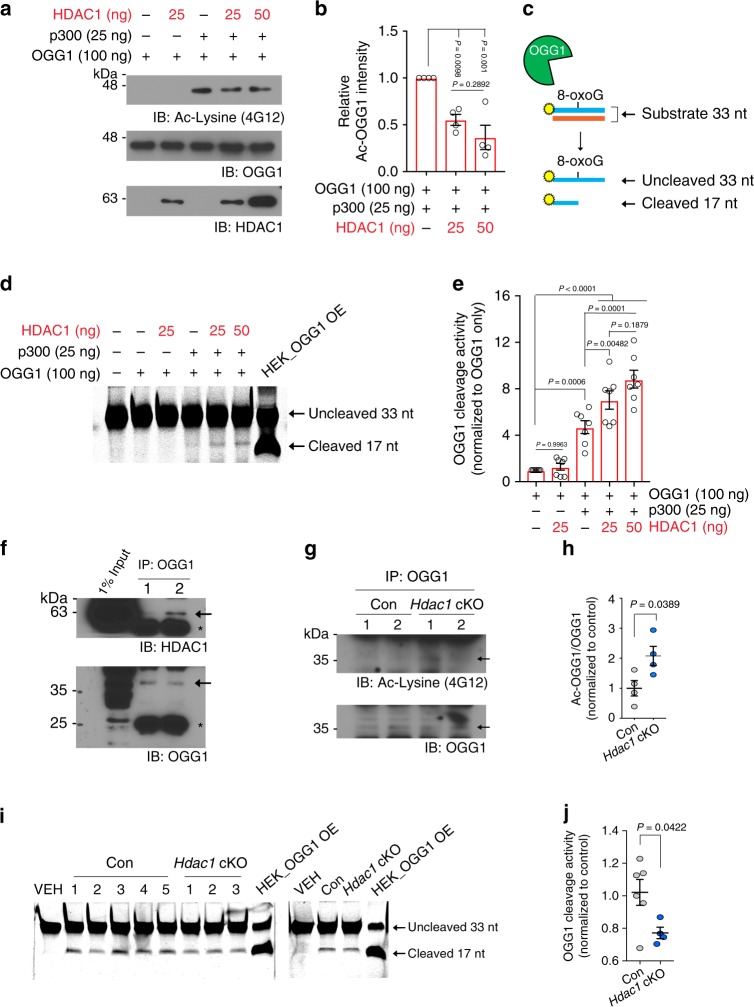
HDAC1 modulates OGG1 activity. **a**, **b** Representative images and quantification of OGG1 acetylation assessed by western blot analysis using antibody against acetylated lysine (Ac-Lysine, 4G12). Recombinant OGG1 (100 ng) was first acetylated by p300 (25 ng), and then incubated with titrated amount of HDAC1 (25 and 50 ng). **a**, **b** Four replicates from four independent experiments. **c** Reaction scheme of OGG1 cleavage assay. 5′IRDye800CW-labeled oligonucleotides (33 nt) that contain an 8-oxoG base at position 17 and complementary unlabeled strand were used as OGG1 substrate. Recombinant OGG1 proteins were incubated with OGG1 substrate and resolved by 20% denaturing urea PAGE. **d**, **e** Representative images and quantification of OGG1 cleavage activity on the reaction samples shown in Fig. 3a. **d**, **e** Seven replicates from four independent experiments. Nuclear extracts from HEK cells overexpressing OGG1 were used as a positive control. **f** Co-immunoprecipitation of OGG1 with HDAC1 in cortical nuclear extracts from 2-month-old wild-type mice (*n* = 2) from one experiment. The asterisks indicate the nonspecific cross-reaction with the IgG heavy and light chains. **g**, **h** Representative images and quantification of acetylation status of endogenous OGG1 in hippocampal lysates of 13M control (*n* = 4) and *Hdac1* cKO (*n* = 4) animals. **g**, **h** Two independent experiments. **i** Representative images of OGG1 cleavage assay using hippocampal nuclear extracts of 13M control and *Hdac1* cKO mice. **j** Quantification of cleaved 17-nt products after OGG1 cleavage assay using hippocampal nuclear extracts of 13M control (*n* = 6) and *Hdac1* cKO (*n* = 4) animals. **i**, **j** Two independent experiments. All values are shown as mean ± SEM. Statistical analysis: **b**, **e** one-way ANOVA with Tukey’s post hoc test; **h**, **j** two-tailed Student’s *t* test. Source data are provided as a Source Data file.

The base excision repair pathway is responsible for 8-oxoG removal, with OGG1 as the major DNA glycosylase in mammals^7,20^. OGG1 can be acetylated by the histone acetyltransferase (HAT) p300, which stimulates OGG1 activity, as well as deacetylated by HDAC1, presumably on lysine residues acetylated by p300^47^. We explored whether 8-oxoG accumulation in aged *Hdac1* cKO mice could be due to altered OGG1 activity. We acetylated recombinant OGG1 in vitro by incubation with p300. Increased acetylation of OGG1 was detected after p300 incubation (Fig. 3a, b). The acetylation reaction was then terminated by the addition of a p300 inhibitor, followed by the addition of recombinant HDAC1. We found that incubation with HDAC1 significantly reduced OGG1 acetylation, confirming that HDAC1 is capable of directly deacetylating OGG1 (Fig. 3a, b).

We next assessed the effects of HDAC1-mediated deacetylation on OGG1 using an OGG1 cleavage assay, which measures the successful reaction of two sequential steps catalyzed by OGG1, 8-oxoG base removal and incision of apurinic and apyrimidinic (AP) sites from a fluorophore-labeled OGG1 substrate^48–50^. Active OGG1 cleaves at the 8-oxoG base of the full-length substrate to generate a 17-nt fluorophore-labeled oligonucleotide (Fig. 3c). As reported previously^47^, incubation with p300 stimulated OGG1 cleavage activity (Fig. 3d, e). Interestingly, HDAC1 further enhanced the activity of acetylated OGG1 (Fig. 3d, e), likely at the AP site cleavage step (Supplementary Fig. 3). This stimulatory effect of HDAC1 on OGG1 was dependent on p300-mediated acetylation (Fig. 3d, e) and sensitive to HDAC1 heat inactivation and pharmacological inhibition (Supplementary Fig. 4a–d). Mass spectrometry analysis identified p300-acetylated lysine residues on OGG1 exhibiting both decreased and increased acetylation by the addition of HDAC1, indicating a complex interplay between p300 and HDAC1 in mediating OGG1 activity (Supplementary Fig. 5). Nonetheless, our findings clearly indicate that HDAC1 stimulates OGG1 activity in vitro, likely by deacetylating a specific subset of lysine residues.

We next determined whether HDAC1 interacts with OGG1 and modulates its activity in vivo. OGG1 was co-immunoprecipitated by HDAC1 from cortical and hippocampal nuclear extracts prepared from wild-type animals (Fig. 3f, Supplementary Fig. 6), confirming in vivo interaction. Elevated 8-oxoG lesions in aged *Hdac1* cKO mice (Fig. 2e, f) were paralleled by increased OGG1 acetylation and reduced OGG1 activity in hippocampal nuclear extracts from these animals, despite similar overall OGG1 levels (Fig. 3g–j; Supplementary Fig. 4e, f). Collectively, these results indicate that HDAC1 physically interacts with OGG1 and contributes positively to OGG1 activity in the aging brain.

### HDAC1 alters 8-oxoG levels and gene expression in 5XFAD mice

**Fig. 4:**
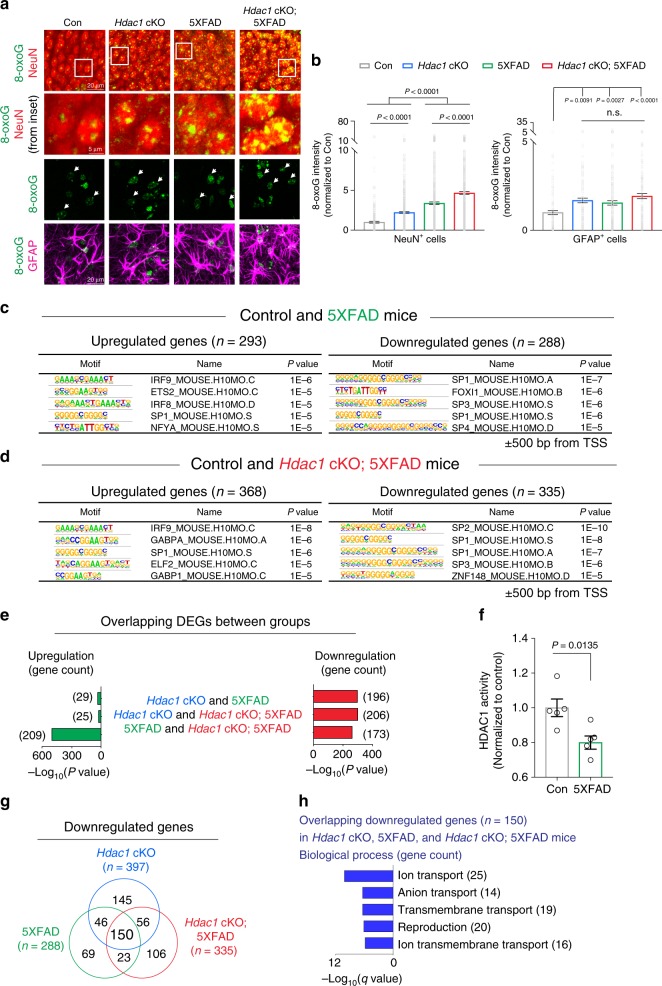
Increased 8-oxoG lesions and downregulation of genes with guanine-rich motifs at promoters in 5XFAD mice. **a**, **b** Representative images and quantifications of 8-oxoG intensity in NeuN^+^ cells and GFAP^+^ cells. 14M animals stained for 8-oxoG (green), NeuN (red), and GFAP (magenta). **a**, **b** Three mice were analyzed per group (two sections/mouse). Nuclei analyzed were: NeuN^+^ cells, 409 (control), 683 (*Hdac1* cKO), 962 (5XFAD), 910 (*Hdac1* cKO; 5XFAD); GFAP^+^ cells, 104 (control), 121 (*Hdac1* cKO), 204 (5XFAD), 155 (*Hdac1* cKO; 5XFAD). White arrows indicate GFAP^+^ cells. **c** Known motifs identified by HOMER program^40^ within ±500 bp from TSS of DEGs **c** 13M control and 5XFAD mice. **d** 13M control and *Hdac1* cKO; 5XFAD mice. **e** Bar graphs showing the amount of DEGs overlaps between groups (*Hdac1* cKO, 5XFAD, and *Hdac1* cKO; 5XFAD mice at the age of 13 months) and enrichment significance. **f** Hippocampal tissues from 13M control and 5XFAD mice were assayed for HDAC1 activity. Five animals were analyzed in each group. **g** A Venn diagram showing the overlap in downregulated genes between *Hdac1* cKO, 5XFAD, and *Hdac1* cKO; 5XFAD mice at the age of 13 months. **h** Enriched GO terms of overlapping genes between *Hdac1* cKO, 5XFAD, and *Hdac1* cKO; 5XFAD mice at the age of 13 months, annotated using MSigDB^31,32^. All values are shown as mean ± SEM. Statistical analysis: **b** one-way ANOVA with Tukey’s post hoc test, n.s., not significant. *P* value of each comparison: *Hdac1* cKO versus 5XFAD = 0.8823; *Hdac1* cKO versus *Hdac1* cKO; 5XFAD = 0.5983; 5XFAD versus *Hdac1* cKO; 5XFAD = 0.1215; **c**, **d** cumulative binomial distributions; **e** two-tailed Fisher’s exact test; **f** two-tailed Student’s *t* test; **h** weighted Kolmogorov–Smirnov-like test followed by multiple hypothesis testing. Source data are provided as a Source Data file.

Elevated 8-oxoG lesions have been reported in AD patients^51^. To experimentally examine oxidative damage in an AD mouse model, and to interrogate the roles of HDAC1, we crossed *Hdac1* cKO to 5XFAD mice, a widely used transgenic model that expresses mutated forms of the human *APP* and *PSEN1* genes^21^. We determined 8-oxoG levels in 5XFAD mice deficient for HDAC1 by co-labeling 8-oxoG with cell-type-specific makers in aged mouse brains. Compared to controls, 8-oxoG intensity was elevated in *Hdac1* cKO and 5XFAD mice in hippocampal neurons and astrocytes (Fig. 4a, b). However, neuronal 8-oxoG intensity was even higher in compound transgenic mice (*Hdac1* cKO; 5XFAD mice) (Fig. 4a, b), suggesting that loss of HDAC1 exacerbates 8-oxoG lesions in hippocampal neurons of 5XFAD mice.

We next conducted RNA-seq on 13-month-old hippocampal tissues to assess whether HDAC1 deficiency alters gene expression in 5XFAD animals. We found 581 and 703 DEGs in 5XFAD and compound transgenic mice, respectively, compared to controls, with approximately equal numbers of up- and downregulated genes in each case (Fig. 4c, d). In all, 65.7% of genes altered in 5XFAD mice were also affected in compound mice, indicating highly similar transcriptional alterations (Supplementary Fig. 7a). Notably, the promoters of downregulated genes in 5XFAD mice were enriched with similar guanine-rich consensus motifs for the SP family of TFs observed in aged *Hdac1* cKO mice (Fig. 4c, d). Indeed, genes downregulated in aged *Hdac1* cKO mice also exhibited reduced expression levels in 5XFAD and compound transgenic mice (Fig. 4e). These similar gene expression changes were not likely due to shared alterations in cell-type composition (Supplementary Fig. 8). Further, we observed a reduction in hippocampal HDAC1 activity in aged 5XFAD mice, but no significant difference in p300 activity (Fig. 4f; Supplementary Fig. 7c). These findings implicate HDAC1 dysfunction as a contributor to 8-oxoG accumulation and altered gene expression in 5XFAD mice.

Of the downregulated DEGs in 5XFAD (*n* = 288), compound transgenic (*n* = 335), and *Hdac1* cKO (*n* = 397) mice, 150 were shared by all three groups, representing 38–52% of all downregulated genes in each group (Fig. 4g). Consistent with downregulated DEGs in *Hdac1* cKO mice, these 150 genes function primarily in ion transport pathways (Fig. 4h). In contrast, only 19 of the upregulated genes were common to all three groups (Supplementary Fig. 7b). These results suggest that *Hdac1* cKO and 5XFAD mice both exhibit elevated 8-oxoG lesions, and share a common gene expression signature characterized by the downregulation of transcripts with guanine-rich promoter elements.

We compared DEGs between 5XFAD and compound transgenic group and found 257 genes (149 upregulated and 108 downregulated genes) were differentially expressed in response to HDAC1 deficiency (Supplementary Fig. 7d). The upregulated genes were associated with defense and immune responses, and downregulated genes with regulation of cell activation (Supplementary Fig. 7d). Guanine-rich motifs were less enriched in genes downregulated in compound transgenic mice compared to the other downregulated gene sets examined herein (Supplementary Fig. 7e). These findings suggest that genes sensitive to 8-oxoG lesions were already repressed in 5XFAD mice, potentially due to impaired HDAC1 activity.

### Exifone is an HDAC1 activator in vitro and in vivo

Our results indicate that loss of HDAC1 results in age-dependent cognitive decline, paralleled by reduced OGG1 activity and 8-oxoG lesion accumulation. These findings suggest that enhancing HDAC1 function could provide benefit in both aging and neurodegeneration. Our previous screening efforts to identify small molecule activators of HDAC1 yielded exifone as an HDAC activator and a strong candidate for such a therapeutic strategy (Supplementary Fig. 9a)^52^. Exifone has also been shown to have pro-cognitive effects in animal models and in patients with Alzheimer’s-type dementia and Parkinson’s disease^53–56^.

We tested whether exifone treatment of cultured neurons could reduce DNA DSBs and histone acetylation^15,17^. Exifone treatment led to a 50% reduction in acetylated H4K12 that was largely blocked by *Hdac1* knockdown, indicating the specificity of the effect (Supplementary Fig. 9b–e). In contrast, exifone treatment of cultured astrocytes showed no effect on H4K12 acetylation, suggesting distinct sensitivity or regulation in the two cell types (Supplementary Fig. 9f, g). Exifone treatment reduced etoposide-induced DNA DSB-marker γH2AX in cultured neurons (Supplementary Fig. 9h, i), consistent with our previous reported HDAC1 overexpression^15^.

To test its effects in vivo, we administrated exifone in CK-p25 mice, an inducible neurodegeneration model, characterized by HDAC1 inhibition, increased neuronal DNA DSBs, and memory deficits^14^. CK-p25 mice were induced for 6 weeks, and they received either exifone or vehicle by intraperitoneal injection for the last 3 weeks of the induction period. Exifone was detectable in mouse brains after a single intraperitoneal injection (Supplementary Fig. 10a), indicating effective brain penetration. Compared to the vehicle group, administration of exifone in CK-p25 mice reduced γH2AX-positive cells in the hippocampal CA1 region (Supplementary Fig. 10b, c), consistent with the positive function of HDAC1 in DSB repair. Moreover, exifone treatment enhanced memory function in CK-p25 mice, as evidenced by increased freezing behavior in a contextual fear conditioning test, without alternating locomotor activity (Supplementary Fig. 10d, e). These findings indicate that exifone treatment produces similar effects as HDAC1 overexpression both in vitro and in vivo.

### HDAC1 protects against 8-oxoG lesions in aged and 5XFAD mice

**Fig. 5:**
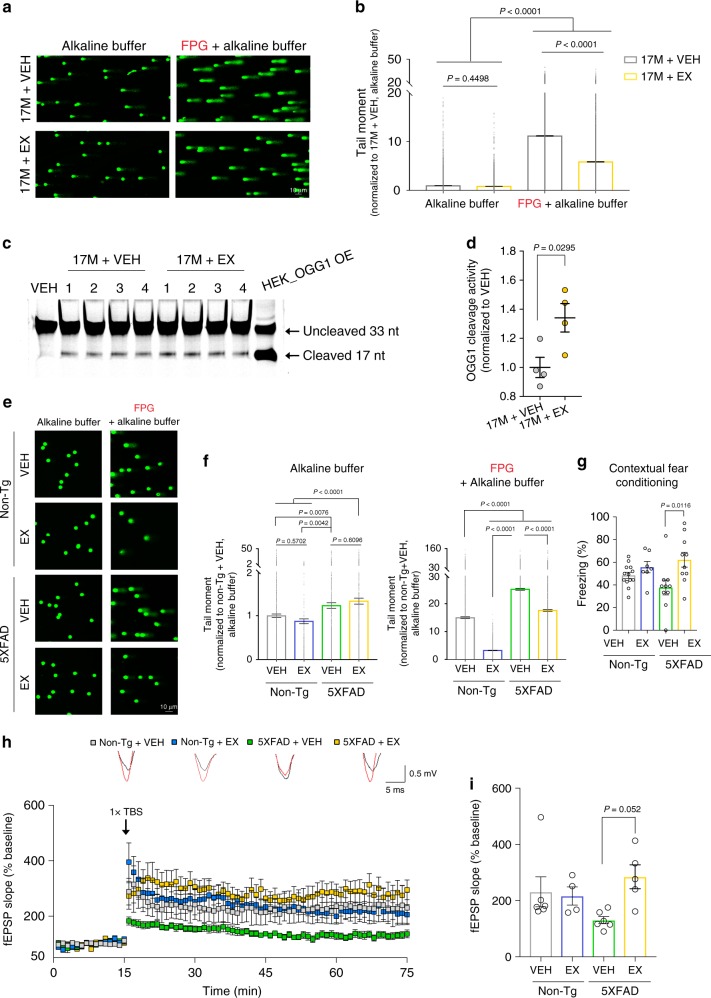
Pharmacological activation of HDAC1 reduces 8-oxoG lesions in aged wild-type and 5XFAD mice. **a**, **b** Representative images and quantification of the comet assay using hippocampal homogenates from 17-month-old (17M) wild-type (WT) mice after vehicle (VEH) or exifone (EX) treatment for 4 weeks. **a**, **b** Nuclei analyzed (three mice/group) were: alkaline, 4746 (17M + VEH), and 3702 (17M + EX); FPG + alkaline, 6254 (17M + VEH), and 4268 (17M + EX). **c** Hippocampal nuclear extracts from 17M WT mice that received daily treatment of either VEH or EX for 4 weeks were used to assess OGG1 cleavage activity. **d** Quantification of cleaved 17-nt products presented in **c**. **c**, **d** Four animals in each group were analyzed from one experiment. **e** Representative images and quantification plots of the comet assay using hippocampal homogenates from 8-month-old non-Tg control and 5XFAD mice treated daily with VEH or EX for 2 weeks. **e**, **f** Nuclei analyzed (at least three mice/group) were: alkaline, 6046 (non-Tg + VEH), 1385 (non-Tg + EX), 3140 (5XFAD + VEH), 3227 (5XFAD + EX); FPG + alkaline, 4177 (non-Tg + VEH), 2030 (non-Tg + EX), 4179 (5XFAD + VEH), and 3075 (5XFAD + EX). **g** Analysis of freezing behavior during contextual fear conditioning test. Animals analyzed were: 13 (non-Tg + VEH), 7 (non-Tg + EX), 10 (5XFAD + VEH), and 10 (5XFAD + EX). **h**, **i** Hippocampal LTP induction in 14-month-old non-Tg control and 5XFAD mice treated daily with VEH or EX for 2 weeks. Animals/slices analyzed in each group were: 3/6 (non-Tg + VEH), 3/4 (non-Tg + EX), 3/6 (5XFAD + VEH), and 3/5 (5XFAD + EX). Overlay of the representative fEPSPs before (black trace) and after (red trace) LTP induction. All values are shown as mean ± SEM. Statistical analysis: **b**, **f**, **g**, **i** one-way ANOVA with Tukey’s post hoc test; **d** two-tailed Student’s *t* test. Source data are provided as a Source Data file.

We next asked if pharmacological activation of HDAC1 could enhance OGG1 activity by examining 8-oxoG lesions that accumulate during physiological aging^42^. We treated 17-month-old wild-type mice with exifone (50 mg/kg body weight) daily for 4 weeks by intraperitoneal injection. Exifone had no effect on non-oxidative DNA damage, as shown by the similar tail moment between exifone- and vehicle-treated aged mice in the alkaline comet assay (Fig. 5a, b). By contrast, the FPG-modified comet assay revealed a marked reduction of 8-oxoG lesions in exifone-treated aged mice (Fig. 5a, b). Consistently, there was enhanced OGG1 activity in hippocampal nuclear extracts of exifone-treated aged mice (Fig. 5c, d). Thus, pharmacological activation of HDAC1 further supports our conclusion that HDAC1 stimulates OGG1 to repair 8-oxoG lesions.

As accumulation of 8-oxoG lesions has also been reported in AD patients^51^, we next examined whether exifone treatment could reduce 8-oxoG lesions in 5XFAD mice and improve cognitive performance. Eight-month-old 5XFAD and non-Tg animals were treated with exifone or vehicle for 2 weeks by intraperitoneal injection before testing. Consistent with our immunostaining results (Fig. 4a, b), we detected a higher tail moment in vehicle-treated 5XFAD animals in the FPG-modified comet assay compared to vehicle-treated controls (Fig. 5e, f). Interestingly, exifone-treated animals exhibited reduced tail moment compared to those treated with vehicle, whereas exifone treatment did not reduce tail moment in the alkaline comet assay. Together, these findings suggest that HDAC1 activation reduces 8-oxoG lesions in both wild-type and 5XFAD animals, and suggest the therapeutic potential of this approach in aging and neurodegeneration.

### HDAC1 activation improves cognition in 5XFAD mice

We then conducted contextual fear conditioning to evaluate hippocampal-dependent memory^27^. We observed increased freezing behavior in exifone- compared to vehicle-treated 5XFAD mice, indicating that exifone improves memory without effect on locomotor activity (Fig. 5g; Supplementary Fig. 10f). Moreover, exifone treatment enhanced hippocampal LTP induction in 14-month-old 5XFAD mice relative to vehicle-treated mice (Fig. 5h, i). No improvement in fear memory and LTP induction was observed in exifone-treated non-Tg animals (Fig. 5g–i).

We also delivered exifone directly into mouse brain by intracerebroventricular (ICV) injection to rule out indirect effects of exifone in the periphery due to intraperitoneal delivery. Sixteen-month-old 5XFAD animals were infused with exifone (100 ng/day) for 2 weeks before subjecting them to the Morris water maze test. Compared to the vehicle group, exifone (ICV)-treated animals spent more time in the target quadrant during the probe trial, suggesting an improvement of spatial memory (Supplementary Fig. 11a–c). Similarly, the level of 8-oxoG lesions was reduced in 5XFAD mice that received exifone (ICV), as shown by lower tail moment in the FPG-modified comet assay (Supplementary Fig. 11d, e). These findings are consistent with our observations in 5XFAD animals receiving exifone treatment intraperitoneally, and suggest that the beneficial effects of exifone are not likely due to molecular relay from activating peripheral HDAC1. While these results indicate that activation of HDAC1 in the brain by exifone likely mediates the various observed effects, we cannot rule out indirect or off-target effects. Together, our findings show that HDAC1 activation reduces 8-oxoG lesions and enhances memory function and LTP induction in 5XFAD mice, suggesting a promising strategy to combat the pathologies associated with aging, AD, and potentially other neurodegenerative disorders.

### HDAC1 counters oxidative stress-induced gene repression

**Fig. 6:**
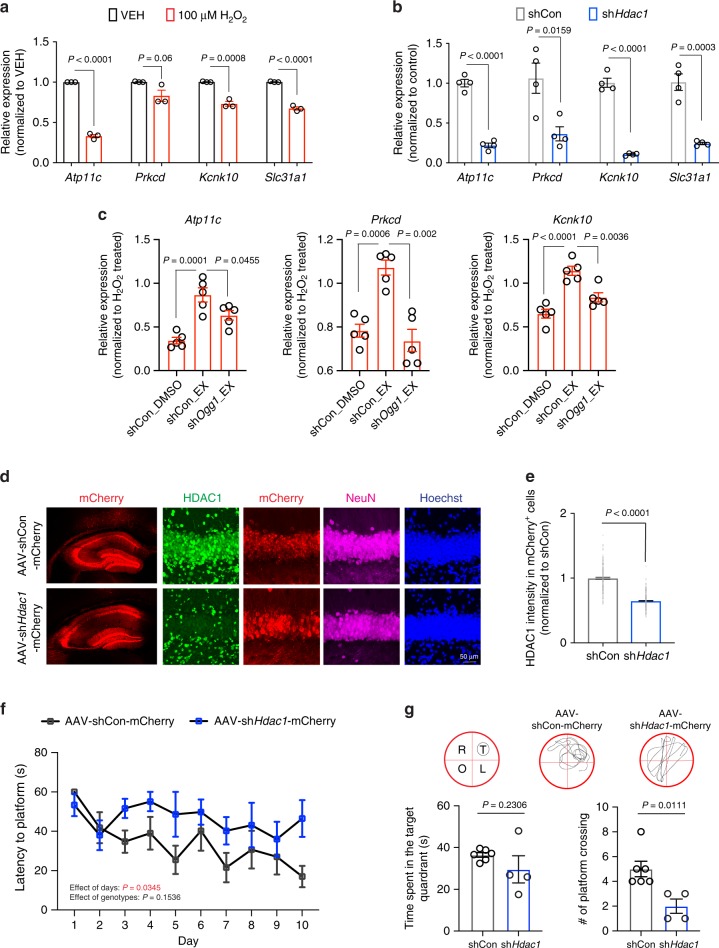
HDAC1 alleviates oxidative damage-mediated gene repression in neurons and neuronal HDAC1 modulates cognition in aged mice. **a** The effect of oxidative damage on gene repression in wild-type cultured neurons (14 DIV) treated with 100 μM H_2_O_2_ overnight. Six replicates from three independent experiments. **b** Gene expression changes in cultured neuron (14 DIV) expressing sh*Hdac1* or scrambled (shCon). Four replicates from four independent experiments. **c** The effects of oxidative damage on gene repression in cultured neurons (14 DIV) expressing sh*Ogg1* or scrambled (shCon) that were preincubated with 1 μM exifone (EX) or vehicle (VEH) for 6 h prior to the addition of H_2_O_2_. Five replicates from five independent experiments. **d** Representative images of the hippocampal region showing HDAC1 levels in aged wild-type mice injected with AAV viruses expressing shRNA against *Hdac1* (AAV-sh*Hdac1*-mCherry) or scrambled (AAV-shCon-mCherry). mCherry (red), HDAC1 (green), NeuN (magenta), and nuclei Hoechst (blue). **e** Quantification showing the HDAC1 intensity in mCherry^+^ cells. **d**, **e** Three mice (two sections/mouse) were analyzed. Cells analyzed were: 197 (AAV-shCon-mCherry), 251 (AAV-sh*Hdac1*-mCherry). **f**, **g** AAV-sh*Hdac1*-mCherry and AAV-shCon-mCherry-injected mice were subjected to the MWM task 3 weeks after viral injection. Shown are the latency to hidden platform during the training, and time spent in each quadrant and number of platform’s crossing during the probe trial. Images showing the location of each quadrant and swim trace of mouse during the probe trial. T = target, O = opposite, R = right, L = left. Animals analyzed were: 6 (AAV-shCon-mCherry) and 4 (AAV-sh*Hdac1*-mCherry). All values are shown as mean ± SEM. Statistical analysis: **a**, **b**, **e**, **g** Two-tailed Student’s *t* test; **c** one-way ANOVA with Tukey’s post hoc test; **f** two-way repeated-measures ANOVA with Bonferroni’s post hoc test. Source data are provided as a Source Data file.

Our findings demonstrate that HDAC1 deficiency leads to reduced OGG1 activity, concomitant with accumulated 8-oxoG lesions and aberrant transcriptional repression of genes involved in brain function. To test whether HDAC1 can confer neuronal protection against oxidative damage-mediated gene repression, we exposed cultured neurons to a mild oxidative stress that increases 8-oxoG lesions^4^ without reducing neuronal survival (Supplementary Fig. 12a). First, we found that the downregulated genes in aged *Hdac1* cKO mice (see Fig. 2b) exhibited reduced expression in neurons exposed to this mild oxidative stress (Fig. 6a). Moreover, the expression of these genes was also decreased in neurons expressing small hairpin (sh) against *Hdac1* (Fig. 6b, Supplementary Fig. 12b). Thus, similar to aged *Hdac1* cKO mice, cultured neurons with either HDAC1 depletion or exposed to mild oxidative stress showed repression of genes with guanine-rich promoters. Consistently, pre-incubating cultured neurons with exifone alleviated oxidative damage-induced gene repression (Fig. 6c). Furthermore, knocking down *Ogg1* attenuated the effect of HDAC1 activation on oxidative damage-induced gene repression (Fig. 6c, Supplementary Fig. 12c). Together, these findings support our conclusion that activation of HDAC1 counteracts oxidative damage-mediated gene repression in an OGG1-dependent manner.

### Neuronal HDAC1 depletion in aged mice impairs memory

Given that HDAC1 was depleted in both neurons and astrocytes in *Hdac1* cKO mice, we next assessed whether knockdown of HDAC1 in neurons was sufficient to cause cognitive deficits in aged animals. We injected bilaterally into the hippocampus of aged wild-type animals an adeno-associated virus (AAV) subtype, PHP.eB, that preferentially transduces neurons^57^ and expresses either scrambled or *Hdac1* shRNA with an mCherry reporter. Reduced HDAC1 immunoreactivity in animals that received sh*Hdac1* demonstrated successful knockdown (Fig. 6d, e). These mice were subjected to Morris water maze test for memory assessment 3 weeks after viral injection. We observed no significant difference in spatial learning during the acquisition phase of the Morris water maze task. However, aged mice that received sh*Hdac1* exhibited a lower number of platform crossings in the probe trial (Fig. 6f, g), indicative of memory deficits. Analysis of the total distance traveled and latency to visible platform showed the cognitive deficits observed were not due to reduced swimming velocity or impaired vision (Supplementary Fig. 13). Thus, reduced neuronal expression of HDAC1 is sufficient to impair memory function in aged mice.

## Discussion

HDAC1 deficiency resulted in astrogliosis, increased GFAP immunoreactivity, and astrocytic hypertrophy in aged mice. Astrocytes adopt morphological changes under stress, injury, and neurodegenerative conditions^58^. Thus, the alterations observed in aged *Hdac1* cKO mice could be due to increased 8-oxoG levels in astrocytes lacking HDAC1 and/or to other changes affecting the brain environment. Astrocytes modulate synaptic plasticity and memory function^59^. Transcriptomic analysis of astrocytes reveals changes in pathways that potentially affect cognition in aged *Hdac1* cKO mice, including lipid metabolism^60^ and immune-related responses^61^ (Supplementary Fig. 14). These observations suggest potential contributions of astroglial HDAC1 to cognitive function in aged animals.

Unlike 2-month-old animals, 6-month-old *Hdac1* cKO mice already exhibit impaired hippocampal LTP induction. Whether hippocampal LTP induction is further impaired in 13-month-old *Hdac1* cKO animals remains to be addressed, due to technical difficulty of this measurement in aged animals. It is possible that the deficits in LTP induction and cognition in *Hdac1* cKO could be attributable to neuronal as well as astrocytic dysfunction.

DNA DSBs is considered the most deleterious type of DNA damage in neurons^7^; however, their incidence is lower than other types of DNA lesions^62^. The brain having high energy consumption is constantly exposed to dangerous ROS species and consequently ROS-associated damage. In neurodegenerative diseases, ROS levels are further elevated, resulting in increased 8-oxoG lesions and mutations that jeopardize genome integrity^62^. These studies, and our findings, suggest that 8-oxoG lesions may be one of the major types of DNA damage that contribute to neurodegenerative phenotypes, underscoring the importance of HDAC1 function in brain health.

Our findings provide a possible link between oxidative DNA damage and impairment of neuronal and cognitive function. We show that the downregulated genes shared by aged *Hdac1* cKO and 5XFAD mice primarily function in ion transport pathways, and are also downregulated in aged humans and AD patients^63,64^. Disruptions of ion transport contribute to ionic gradient imbalance and altered neuronal excitability, two pathological features of brain aging and neurodegeneration^65,66^. These findings indicate that 8-oxoG accumulation and downregulation of ion transport may contribute to functional decline in aged *Hdac1* cKO and 5XFAD mice. Further, we show that exifone treatment reduces 8-oxoG levels and enhances cognition in 5XFAD animals with minimal effects on tail moment in the alkaline comet assay, suggesting it acts primarily through reducing 8-oxoG lesions.

One intriguing observation was the significant overlap of downregulated genes with guanine-rich promoters between aged *Hdac1* cKO and 5XFAD mice, coincident with the elevated 8-oxoG lesions and reduced HDAC1 activity in 5XFAD mice. While the mechanisms underlying impaired HDAC1 function remain to be confirmed, the effect is likely mediated by the p25/CDK5 complex. HDAC1 activity is inhibited in CK-p25 mice that overexpress p25, an activator of CDK5 kinase function. p25 is overexpressed in multiple neurodegenerative diseases and disease models, including 5XFAD mice^21^, suggesting that HDAC1 inhibition and increased 8-oxoG lesions are likely mediated via a common pathological mechanism involving p25/CDK5 dysregulation.

Besides OGG1, two other DNA glycosylases (NEIL1 and NTH1) possess 8-oxoG processing capacity; however, both have weaker substrate preference^67^, and their depletion did not result in 8-oxoG accumulation in aged mouse brains^68^. OGG1 is regulated by a complex interplay between acetylation and deacetylation, affecting catalytic activity and protein stability^69,70^. OGG1 is acetylated by p300 on multiple sites, promoting its activity^47^. The effects of OGG1 deacetylation appear to be more complex, with both increased and decreased activation and stability^69,70^. Our work demonstrates that HDAC1 is an OGG1 deacetylase in the brain, likely stimulating OGG1 activity by promoting AP site incision. Our findings link HDAC1 and OGG1 to oxidative DNA damage, neuronal gene expression, synaptic plasticity, and cognitive performance in brain aging and neurodegenerative disease, and highlight the therapeutic potential of pharmacological HDAC1 activation in aging and neurological disorders.

## Methods

### Mouse strains

All mouse work was approved by the Committee for Animal Care of the Division of Comparative Medicine at Massachusetts Institute of Technology. *Hdac1*^*f/f*^ mice were gifted from E.N. Olson^23^. *Hdac1*^*f/f*^ mice were bred with *Nestin-Cre* transgenic mice^24^ to generate *Hdac1* brain-specific conditional knockout (*Hdac1* cKO) mice. 5XFAD mice were obtained from Jackson Laboratory (JAX ID# 34840), and were crossed with *Hdac1* cKO mice to generate *Hdac1* cKO; 5XFAD (compound transgenic) mice. Aged C57BL/6J mice were obtained from National Institute on Aging. Two- to three-month-old male mice were used as young group, and 13- to 17-month-old male mice were used as aged group. Mice were housed in groups of three to five animals on a standard 12 h light/12 h dark cycle, temperature (24 °C), and humidity (45%) with food and water available ad libitum. All experiments were performed during the light cycle.

### Antibodies

The antibodies used for immunostaining were as follows: anti-HDAC1 (Thermo, PA1-860, 1:500), anti-GFAP (Abcam, ab53554, 1:500), anti-NeuN (Rbfox3) (Synaptic Systems, 26604, 1:500), anti-IBA1 (Wako Chemicals, 019-19741, 1:500), cleaved caspase-3 (Abcam, ab2302, 1:200), anti-8-OHdG N45.1 (Cosmo Bio, NNS-MOG-020-EX, 1:100), and anti-γH2AX (Millipore, 05-636, 1:500). The antibodies used for western blots were as follows: anti-HDAC1 (Abcam, ab7028, 1:2000), anti-OGG1 (Novus Biologicals, NB100-106, 1:1000), anti-Lamin B1 (Abcam, ab16048, 1:1000), anti-GAPDH (Cell Signaling Technology, 2118, 1:5000), anti-acetyl-lysine 4G12 (Millipore, 05-515, 1:2000), anti-γH2AX (Millipore, 05-636, 1:2000), anti-acetyl-histone H4 (Lys12) (Millipore, 07-595, 1:2000), anti-histone H3 (Abcam, ab24834, 1:10,000), and p300 (Thermo Fisher, MA1-16608, 1:1000). Antibodies used for immunoprecipitation and ChIP experiments were anti-OGG1/2 (Santa Cruz Biotechnology, sc-376935), anti-OGG1 (Novus Biologicals, NB100-106), anti-HDAC1 (Cell Signaling Technology, 5356), p300 (Thermo Fisher, MA1-16608), and anti-8-OHdG N45.1 (Cosmo Bio, NNS-MOG-020-EX).

### Immunohistochemistry analysis

Mice were transcardially perfused with cold phosphate-buffered saline (PBS), followed by perfusion with cold 4% paraformaldehyde in PBS. Brains were post fixed in 4% paraformaldehyde at 4 °C overnight, and prepared as 40-μm-thick vibratome coronal sections in PBS. Antigen retrieval was performed for HDAC1 immunostaining. Brain sections were immersed in citrate buffer (10 mM sodium citrate, 0.05% Tween-20, pH 6.0 in PBS) at 80 °C for 20 min, and cooled to room temperature (RT) for 1 h. Free-floating sections were next permeabilized and blocked in PBS containing 0.2% Triton X-100 and 10% normal donkey serum at RT for 1 h, and then incubated overnight at 4 °C with primary antibodies diluted in blocking buffer. Primary antibodies were visualized using the appropriate secondary antibodies conjugated to Alexa Fluor 488, Alexa Fluor 594, and Alexa Fluor 647 antibodies (Thermo Fisher Scientific, all diluted 1:500 in blocking buffer) and nuclei were visualized with Hoechst 33342 (Thermo Fisher Scientific, H1399). Samples were washed three times in PBS each after incubation with primary and secondary antibodies. Images were acquired on a Zeiss LSM710 confocal microscope. Quantification of GFAP and γH2AX immunoreactivity was performed with the ImageJ software (version 1.51m9). Cell size of astrocytes, 8-oxoG intensity in aged mouse brain, and HDAC1 intensity of mCherry^+^ cells were analyzed with the Imaris image analysis software (Bitplane). For each experiment, two coronal sections from at least three animals were used for quantification.

### Western blotting

Cultured neurons were washed once with cold 1× PBS and lysed in RIPA buffer (150 mM NaCl, 1% IGEPAL, 0.5% NaDOC, 0.1% SDS, and 50 mM Tris-HCl, pH 8.0, supplemented with protease inhibitors). The supernatant was collected after centrifugation at 12,000 × *g* for 10 min at 4 °C. For brain tissue, nuclear fractions were extracted using the Subcellular Protein Fractionation Kit for Tissue according to the manufacturer’s instructions (Thermo Scientific, 87790). Lysates were quantified using Bio-Rad Protein Assay, and equal amount of protein was loaded onto a 10% acrylamide gels. Protein was transferred from the gel to 0.45 μm PVDF membranes (Millipore) at 100 V for 2 h and blocked with 5% non-fat milk in TBST (10 mM Tris-HCl pH 8.0, 150 mM NaCl, 0.05% Tween-20) for 1 h before application of primary antibodies. Membranes were incubated with primary antibodies overnight at 4 °C and secondary antibodies at RT for 1 h. Proteins were detected by a Chemiluminescent Kit and visualized by an autoradiographic film. Western blots were analyzed with the ImageJ software (version 1.51m9).

### RNA extraction and qPCR

Hippocampus was snap frozen in liquid nitrogen and stored at −80 °C, and total RNA was extracted using TRIzol reagent (Invitrogen) according to the manufacturer’s instructions. Reverse transcription of total RNA (1 μg) was carried out using RNA to cDNA EcoDry Premix (Oligo dT) (Clontech) according to the manufacturer’s protocol. qPCR was performed using a Bio-Rad CFX-96 quantitative thermocycler and SsoFast EvaGreen Supermix (Bio-Rad). Relative changes in gene expression were determined using the 2^−ΔΔCt^ method. Primer sequences used for qPCR can be found in Supplementary Table 1.

### Behavioral experiments

Mice were handled more than 3 days prior to behavioral tests, and were left to acclimate in the testing rooms for at least 1 h before experiments. For open field test, mice were placed in the center of an open field box (40 cm × 40 cm × 30 cm), and habituated for 10 min. Activity was measured automatically by the VersaMax software (AccuScan Instruments) as movement across a grid of infrared light beams during habituation. Morris water maze task was conducted in a circular tank (1.2 m diameter) filled with opaque water at 22 °C with spatial reference cues on the wall surrounding it. The tank contained a hidden fixed platform (10 cm diameter) in a target quadrant. During training, mice were randomly placed into water maze facing the wall of the tank, and allowed to search for the hidden platform for 60 s. Mice were guided to the platform and kept on the platform for 30 s if they failed to locate it within 60 s. Two trials a day were conducted, and mice were kept on a heating pad between trials. One day after the last training, the platform was removed to conduct a probe trial for assessing spatial memory. Mouse behavior was video recorded and analyzed using EthoVision XT (Noldus Information Technology). Latency to hidden platform during training, time spent in each quadrant, and total distance traveled during probe trial (60 s) were recorded and analyzed automatically. The number of platform crossing during the probe trial was analyzed manually. Visible platform test was performed 1 h after probe trial to assess visual deficiency. For contextual fear conditioning test, mice were placed in a conditioning chamber with acrylic walls and a metal grid bottom that delivered a foot shock (TSE Systems). After 3 min of habituation, mice received a foot shock (2 s, 0.4–0.8 mA constant current) and remained in the context for an additional 30 s before returning to their home cage. Contextual fear memory was measured 24 h after foot shock by scoring freezing behavior automatically for 3 min in the context used during conditioning. Each behavioral test assessing cognitive function (fear conditioning and Morris water maze test) was conducted using different cohort of animals. The number of cohorts of animals for each behavioral task was as follows: one cohort of animals for Figs. 1e–g, 6f, g, Supplementary Fig. 1g–i, Supplementary Fig. 10d, e and Supplementary Fig. 11a–c; two cohorts of animals for Fig. 5g and Supplementary Fig. 10f.

### Single-cell electrophoresis (comet) assay

Hippocampal tissues were homogenized in 0.5 mL cold PBS using a rotor stator on ice. After homogenization, cells were run through a 0.45 μm cell strainer and diluted with cold PBS to 4 × 10^5^ cells/mL. Fifty microliters of diluted cells were added to 450 μL of 1% low melting point agarose (Invitrogen) kept at 42 °C and applied onto each well of comet slides (Trevigen, 4250-004-03). Comet slides were kept at 4 °C in the dark for 30 min, and then immersed in cold lysis solution (Trevigen, 4250-050-01) that contained 10% dimethyl sulfoxide (DMSO) at 4 °C in the dark for 14 to 16 h. After overnight lysis, slides were incubated with freshly prepared pre-chilled alkaline buffer (300 mM NaOH, 1 mM EDTA) for 1 h in the dark at 4 °C, and then subjected to electrophoresis in 850 mL cold alkaline buffer using CometAssay® Electrophoresis System II (Trevigen, 4250-050-ES) following the manufacturer’s instructions. For FPG treatment, slides were digested with FPG enzyme (NEB, M0240, 1:1000) in a reaction buffer (40 mM HEPES, 0.1 M KCl, and 0.2 mg/mL bovine serum albumin (BSA), adjust to pH 8.0 with KOH) at 37 °C for 45 min. Slides were washed two times with cold reaction buffer for 5 min at RT, and subjected to electrophoresis. After electrophoresis, slides were washed with cold neutralization buffer (0.4 M Tris-HCl, pH 7.4) for 15 min twice in the cold room, and then incubated with 70% ethanol at RT for 30 min. Slides were next dried at 37 °C for 30 min to bring cells into the sample plane, and stained with SYBR Gold (Invitrogen, S11494) for 30 min at RT. Slides were imaged using a Zeiss LSM710 confocal microscope (×5 objective) with tile scan, which allowed us to acquire 992 nuclei in average for each animal in this study. Comet images were analyzed using default settings in the OpenComet software, a free plugin for ImageJ (http://www.cometbio.org/index.html).

### Cell culture and cell viability assay

Primary cortical neurons were cultured from Swiss–Webster embryos (embryonic day 16), and then were maintained in neurobasal media (Gibco, 21103) supplemented with l-glutamine (5 mM), penicillin and streptomycin, and B27 neuronal additive. Primary astrocytes were cultured from Swiss–Webster pups (postnatal day 0), and then were maintained in Dulbecco’s modified Eagle’s media (Gibco, 10566) supplemented with l-glutamine (5 mM), penicillin and streptomycin, and fetal bovine serum. Cell viability was determined by the CellTiter-Glo® Luminescent Cell Viability Assay (Promega).

### In vivo drug administration and LC/MS/MS analysis

For intraperitoneal injection, mice received daily dose of 50 mg/kg body weight exifone. Exifone (TCI America, H1065) was dissolved in 10% DMSO, 10% Tween-80, and 80% saline to make 10 mg/mL solution and stored at −20 °C. For cannula implantation, mice were anesthetized with isoflurane in the stereotaxic frame for the entire surgery and their body temperature was maintained using a heating pad. A cannula (26 gauge and 2.5 mm length below pedestal) was implanted into the lateral ventricle (A/P: −0.3 mm; M/L: 0.9 mm; D/V: −2.5 mm relative to the Bregma) and secured by dental cement. Exifone (100 ng) was infused into the lateral ventricle daily for 2 weeks prior to behavioral assessments. On the day of behavioral test, drug infusion was performed 2 h prior to administration of behavioral tests. For liquid chromatography with tandem mass spectrometry (LC/MS/MS) analysis, brain samples were collected from a set of three mice at each time point. Brain samples were homogenized using ice-cold PBS (pH 7.4) in a ratio of 2 (buffer):1 (brain), and homogenates were stored below −80 °C until analysis. Total homogenate was kept at volume three times that of brain weight. All samples were processed for analysis by protein precipitation using acetonitrile and analyzed with fit-for-purpose LC/MS/MS method.

### RNA-sequencing

Total RNA was extracted from hippocampal tissues using TRIzol reagent (Invitrogen), and 3 μg of total RNA was treated with DNase I (Worthington Biochemical), followed by RNA purification using RNA Clean and Concentrator-5 Kit (Zymo Research) according to the manufacturer’s protocols. Purified RNA was subjected to quality control (Fragment Analyzer). cDNA libraries were prepared using Illumina TruSeq Total RNA Sample Prep Kits (Illumina) and High-Throughput 3′Digital Gene Expression method for 3 and 13 months mouse brain, respectively. Libraries were sequenced on the Illumina HiSeq 2000 platform at the MIT BioMicro Center. The collapsed raw fastq reads were aligned by Tophat2, and further processed by Cufflinks 2.0.0 with UCSC mm9 reference gene annotation to determine transcript abundances. The mean yield per sample was 14,217,977 sequencing reads. A gene was considered differentially expressed with a fold change ≥1.5 and a statistical significance of *P* < 0.05. GO analysis of DEGs was performed using gene set enrichment analysis^31,32^ for GO biological process (MSigDB, Broad Institute). TF-binding motif analysis of DEGs was conducted using the HOMER software^40^. Sequencing datasets are available to the public in the GEO Data Bank under accession numbers GSE115437 and GSE147407. The lists of DEGs can be found in Source Data file.

### Cell-type enrichment of DEGs

To examine the possibility that the similar changes in gene expression in aged *Hdac1* cKO and 5XFAD mice were caused by similarly altered cell composition (e.g., astrogliosis), we took advantage of a published cell-type-specific mouse Brain RNA-seq dataset (https://www.brainrnaseq.org). Genes whose expression were enriched >4-fold in one cell type relative to the others were utilized as markers of that cell type. We overlaid the DEGs identified from *Hdac1* cKO and 5XFAD brains onto these cell-type marker genes, and found no cell-type enrichment for *Hdac1* cKO DEGs. In contrast, we observed a significant enrichment of microglia and astrocyte marker genes among the DEGs from 5XFAD mice (Supplementary Fig. 8a). We also analyzed the expression levels of our DEGs in each of the cell types from this dataset (https://www.brainrnaseq.org, Brain RNA-seq dataset) to examine for possible cell-type enrichment, reasoning that if the DEGs were preferentially derived from a particular cell type, their baseline expression would also be higher in that cell type. We found that the DEGs from 5XFAD mice altered expression in microglia, whereas no cell-type-specific expression differences were observed for DEGs from aged *Hdac1* cKO mice (Supplementary Fig. 8b). These findings indicate that the similar changes in gene expression in aged *Hdac1* cKO and 5XFAD mice were not likely due to shared alterations in cell-type composition.

### Chromatin immunoprecipitation

Hippocampi (two hemispheres) were snap frozen in liquid nitrogen and stored at −80 °C. Frozen tissues were homogenized using a dounce tissue grinder (Wheaton) in cold PBS and fixed in 1% formaldehyde for 10 min at RT, and then quenched with 125 mM glycine for 5 min. Fixed homogenates were washed twice by cold NF1 buffer (10 mM Tris-HCl pH 8.0, 5 mM MgCl_2_, 0.1 M sucrose, 1 mM EDTA pH 8.0, 0.5% Triton X-100, proteinase inhibitor cocktail), and incubated with 10 mL of NF1 buffer at 4 °C for 30 min with shaking to release the nuclei. Fixed nuclei were pelleted down and resuspended in LB3 buffer (10 mM Tris-HCl pH 8.0, 1 mM EDTA pH 8.0, 0.5 mM EGTA pH 8.0, 0.5% (w/v) *N*-lauroylsarcosine sodium salt, proteinase inhibitor cocktail) and chromatin shearing using a Bioruptor Plus (Diagenode, setting medium, 25 cycles of 30 s ON, 30 s OFF). Equal amounts of chromatin (3 μg) were pre-cleared and immunoprecipitated overnight at 4 °C with 5 μg antibodies against HDAC1 (Cell Signaling Technology, 5356), anti-8-OHdG N45.1 (Cosmo Bio, NNS-MOG-020-EX), or normal mouse IgG (Millipore, 12-371). Targeted chromatin was enriched using Dynabeads Protein G (Thermo Fisher Scientific, 10003D) for 4 h, followed by four washes with RIPA buffer (50 mM HEPES pH 7.6, 10 mM EDTA, 0.7% (w/v) sodium deoxycholate, 0.5 M LiCl, 1% NP-40, proteinase inhibitor cocktail) and once with TE buffer (50 mM Tris-HCl pH 8.0, 10 mM EDTA pH 8.0). Immunoprecipitated chromatin was eluted twice in TES buffer (50 mM Tris-HCl pH 8.0, 10 mM EDTA pH 8.0, 1% SDS) at 65 °C for 20 min with agitation, and then reverse crosslinked in a 65 °C water bath overnight. Chromatin was next treated with RNase (20 μg) and proteinase K (40 μg), followed by DNA purification using phenol–chloroform extraction and ethanol precipitation. DNA pellets were resuspended in 10 mM Tris-HCl (pH 8.0) and subjected to ChIP-seq or qPCR analysis. Primer sequences used for ChIP-qPCR can be found in Supplementary Table 2.

### ChIP-sequencing

Briefly, HDAC1 ChIP-seq was performed on hippocampal tissues of 3 months wild-type mice (Jackson Laboratory, C57BL/6J). Sequencing libraries were prepared from ~1 to 5 ng ChIP (or input) DNA. Gel electrophoresis was used to retain library fragments between 300 and 600 bp. Prior to sequencing, libraries were quantified using Qubit (Invitrogen) and quality controlled using Agilent’s Bioanalyzer. Sequencing reads were aligned to the mouse genome assembly (mm9) using BWA aligner (samse option). Duplicate reads were removed by samtools. Bed alignment files were generated using bedtools. Peak calling was conducted using MACS2 peak-finding algorithm, and we identified 6998 peaks (0.05% false discovery rate cut-off). Annotated genes associated with HDAC1 binding was identified by the GREAT software utilizing default settings. MACS2 program was used to generate fold enrichment of ChIP-seq signals against input across the genome (bedgraph format). The mean yield per sample was 15,647,780 40-bp single-end reads, of which 12,979,186 reads were aligned (82.9%). The output bedgraph files were used by HOMER program to generate aggregated ChIP-seq signals around gene TSS (5 kb upstream and downstream). Chromatin states were defined by available ChIP-seq datasets, and enrichment of HDAC1 binding over chromatin states was assessed using the ChromHMM software. Sequencing datasets are available to the public in the GEO Data Bank under the accession number GSE115437.

### In vitro acetylation and deacetylation assays

For the acetylation assay, 100 ng recombinant OGG1 (ProSpec, ENZ-253) was incubated with 25 ng recombinant p300 (BPS Biosciences, 50071) at 30 °C for 1 h in the reaction buffer (50 mM Tris-HCl (pH 8.0), 1 mM DTT, 1 mM PMSF, 0.1 mM EDTA (pH 8.0), 50 nM acetyl-CoA, 10% glycerol), followed by incubating with 10 μM C646 (Tocris) at 37 °C for 30 min to stop p300 activity. The samples described above were next subjected to deacetylation assays, which were incubated with recombinant HDAC1 (25 and 50 ng, BPS Biosciences, 50051) at 37 °C for 1 h in the deacetylation buffer (137 mM NaCl, 2.7 mM KCl, 4 mM MgCl_2_).

### OGG1 cleavage assay and mass spectrometry analysis

OGG1 substrate was a duplex of a 5′ IRDye800CW-labeled oligonucleotide synthesized by Bio-Synthesis (Lewisville, TX) with an 8-oxoG at position 17 (5′ IRDye800CW-ATA CGCATATACCGCTXTCGGCCGATCTCCGAT-3′, X = 8-oxoG), and a non-labeled complementary oligonucleotide (5′-ATCGGAGATCGGCCGACAGCGGTATATGCGTAT-3′). Hippocampal nuclear fractions were extracted using the Subcellular Protein Fractionation Kit for Tissue following the manufacturer’s protocols (Thermo Scientific, 87790) and diluted to 1.5 μg/μL with nuclear extraction buffer supplied in the kit. Hippocampal nuclear extracts (22.5 μg) were incubated with OGG1 substrate at 37 °C for 4 h with agitation in a 20-μL reaction (100 fmol OGG1 substrate, 20 mM Tris-HCl pH 8.0, 1 mM EDTA, 200 mM NaCl, 1 mg/mL BSA, 5% glycerol). After the reaction, samples were denatured in loading dye (95% formamide, 18 mM EDTA, 0.025% SDS, 0.025% bromophenol blue, 0.025% xylene cyanol) and loaded onto a 20% polyacrylamide gel containing 8 M urea at 200 V for 45 min. Gels were visualized using the LI-COR Odyssey imaging system, and OGG1 cleavage activity was determined by the amount of cleaved product using the ImageJ software (version 1.51m9). Samples after in vitro acetylation and deacetylation assays (described above) were incubated with OGG1 substrate (100 fmol) at 37 °C for 1 h. Heat-inactivated HDAC1 (80 °C for 20 min) and pharmacologically inhibited HDAC1 (incubated with 100 nM trichostatin A) were used to assess the stimulatory effect of HDAC1 deacetylase activity on OGG1 activity. For mass spectrometry analysis, recombinant OGG1 was incubated alone, with p300 or p300 followed by HDAC1. All three reactions were subsequently subjected to protein electrophoresis. Gel bands containing OGG1 were excised and were enzymatically digested. The resulting peptides were chromatographically separated and introduced into Orbitrap Elite mass spectrometer (Thermo Fisher) where peptides were mass measured within 3 p.p.m. and subsequently fragmented in a low-resolution ion trap.

### Electrophysiology

Mice were anesthetized with isoflurane and decapitated. Transverse hippocampal slices (400 μm thick) were prepared in ice-cold dissection buffer (in mM: 211 sucrose, 3.3 KCl, 1.3 NaH_2_PO_4_, 0.5 CaCl_2_, 10 MgCl_2_, 26 NaHCO_3_, and 11 glucose) using a Leica VT1000S vibratome. Slices were recovered in a holding chamber containing oxygenated (95% O_2_ and 5% CO_2_) artificial cerebrospinal fluid consisting of (in mM) 124 NaCl, 3.3 KCl, 1.3 NaH_2_PO_4_, 2.5 CaCl_2_, 1.5 MgCl_2_, 26 NaHCO_3_, and 11 glucose for 1 h at 28–30 °C. Field recordings were performed at CA1 by stimulating Schaffer collateral fibers with a bipolar electrode. LTP was induced by a single theta-burst stimulation (TBS) after recording of stable baseline for 15 min. TBS consisted of ten bursts, each containing four pulses at 100 Hz. The slopes of field excitatory post-synaptic potentials (fEPSPs) were measured to quantify the strength of synaptic transmission. The magnitude of LTP was calculated by comparing the average slopes of fEPSPs during the last 10 min of recordings with those recorded before stimulation. A HEKA instrument (EPC10) was used for data acquisition, and data analysis was conducted using pClamp10 (Axon Instruments).

### Immunoprecipitation and enzyme activity assay

For immunoprecipitation, hippocampal tissues were dounce homogenized in IP buffer (50 mM Tris-HCl pH 8.0, 150 mM NaCl, 0.5% Triton X-100, 0.1% NP-40). Lysates were pre-cleared with 30 μL of Protein A Sepharose beads (Sigma-Aldrich) at 4 °C for 30 min and incubated with 5 μg of primary antibody overnight on a tube rotator at 4 °C. Beads were washed three times in washing buffer (20 mM HEPES KOH adjust pH 7.9, 0.1 M KCl, 0.01% NP-40) and boiled in 2× loading buffer for western blot analysis. For HDAC1 and p300 activity assay, hippocampal tissues were lysed in IP buffer and immunoprecipitated with anti-HDAC1 antibody (Abcam, ab7028) or anti-p300 antibody (Thermo Fisher, MA1-16608). The washed beads with bound HDAC1 or p300 were assessed for HDAC or histone acetylase activity using the FLUOR DE LYS® HDAC Fluorometric Activity Assay Kit (Enzo Life Sciences) or HAT Activity Assay Kit (Abcam, ab65352) according to the manufacturer’s instructions. HDAC1 or p300 activity was normalized to input HDAC1 or p300 protein levels.

### AAV production and viral injection

The plasmid expressing sh*Hdac1* or scramble control together with an mCherry reporter was packaged into PHP.eB capsids. Aged wild-type mice were anesthetized with avertin, and a small hole was made in the skull above the hippocampus (relative to Bregma: A/P −2.0 mm and M/L ±1.50 mm relative to the Bregma). A Hamilton syringe with 33-gauge needle was lowered to the hippocampus (D/V: −1.5 mm), where 1 μL of AAV was infused with a flow rate of 100 nL/min into each hippocampus. The needle was withdrawn, following a 10-min post-injection period, and mice were allowed to recover for 4 days. The shRNA sequences can be found in Supplementary Table 3.

### Purification and RNA-seq analyses of GFAP^+^ nuclei

In order to obtain enough material for downstream RNA-seq analysis, frozen cortical tissue was homogenized using a dounce tissue grinder (Wheaton) in 1 mL of cold NF1 buffer (10 mM Tris-HCl pH 8.0, 5 mM MgCl_2_, 0.1 M sucrose, 1 mM EDTA pH 8.0, 0.5% Triton X-100, proteinase inhibitor cocktail), and the suspension was centrifuged at 1600 × *g* at 4 °C for 7 min. Pellets were incubated with 10 mL of NF1 buffer at 4 °C for 30 min with shaking to release the nuclei. Released nuclei were then pelleted down at 1600 × *g* for 7 min and incubated with Alexa Flour APC-conjugated GFAP antibody (Cell Signaling Technology, 3657, 1:200) and Alexa Flour 488-conjugated NeuN (Millipore, MAB377X, 1:200) in cold PBS with 1% BSA and proteinase inhibitor cocktail on a tube rotator at 4 °C for 3 h. Pelleted nuclei were then resuspended in 0.5 mL PBS and were run through a 0.45 μm cell strainer prior to FACS sorting. Cytometry data were analyzed using BD FACSDiva 8.0 and FlowJo V10 (Tree Star, Inc.). RNA of GFAP^+^ nuclei was extracted using the RNeasy Plus Mini Kit (Qiagen, 74134) according to the manufacturer’s protocol. SMART-Seq v4 Ultra Low Input RNA Kit (Clontech, 634890) was used for library preparation. Sequencing datasets are available to the public in the GEO Data Bank under accession number GSE115437. The lists of DEGs can be found in Source Data file.

### Statistics

Results are shown as mean ± SEM. All statistical analysis was conducted using the Prism GraphPad software. Two-tailed unpaired *t* test, two-tailed Fisher’s exact test, one-way analysis of variance (ANOVA), followed by Tukey’s post hoc test, two-way ANOVA followed by Bonferroni post hoc test, and two-way repeated-measures ANOVA followed by Bonferroni post hoc test were used. The data were assumed to distribute normally, but this was not formally tested. No statistical method was used to determine sample sizes, but sample sizes in this study were similar to previous publications^14,15^, except for the comet assay. For the comet assays, at least three animals were used in each group and tile scan images using ×5 objective were acquired to obtain all the comets on the slides. Analysis of GFAP^+^ cell volume, 8-oxoG intensity in aged mouse brain, and HDAC1 intensity in mCherry^+^ cells were performed blind to the conditions of the experiment.

### Reporting summary

Further information on research design is available in the Nature Research Reporting Summary linked to this article.

## Supplementary information

Supplementary Information

Reporting Summary

## Data Availability

Source data underlying Figs. 1b,d–i, 2b, d, f, h, 3, 4b, f, 5b–d, f–i, and 6a–c, e–g and Supplementary Figs. 1a, d, f–i, 2a, b, d, e, 3–6, 7c, 8b, 9b–i, 10c–f, 11–13, and 14b are available as a Source Data file. All other relevant data supporting this study are available from the corresponding author upon reasonable request. Cell-type enrichment of DEGs was using published cell-type-specific mouse dataset (https://www.brainrnaseq.org, Brain RNA-seq dataset). Sequencing datasets are available to the public in the GEO Data Bank under accession numbers GSE115437 and GSE147407.

## Source data

Source Data
