# Supplementary material for: HDAC1 modulates OGG1-initiated oxidative DNA damage repair in the aging brain and Alzheimer’s disease
Source: Nat Commun. 2020 May 18;11:2484. doi: 10.1038/s41467-020-16361-y (PMC7235043; doi:10.1038/s41467-020-16361-y)
Supplement: Supplementary file 1 — Supplementary Information [file 41467_2020_16361_MOESM1_ESM.pdf]

Supplementary Information

HDAC1 modulates OGG1-initiated oxidative DNA damage repair in the aging brain and Alzheimer's disease

Pao et al.

Supplementary data (14 Supplementary Figures and 3 Supplementary Tables)

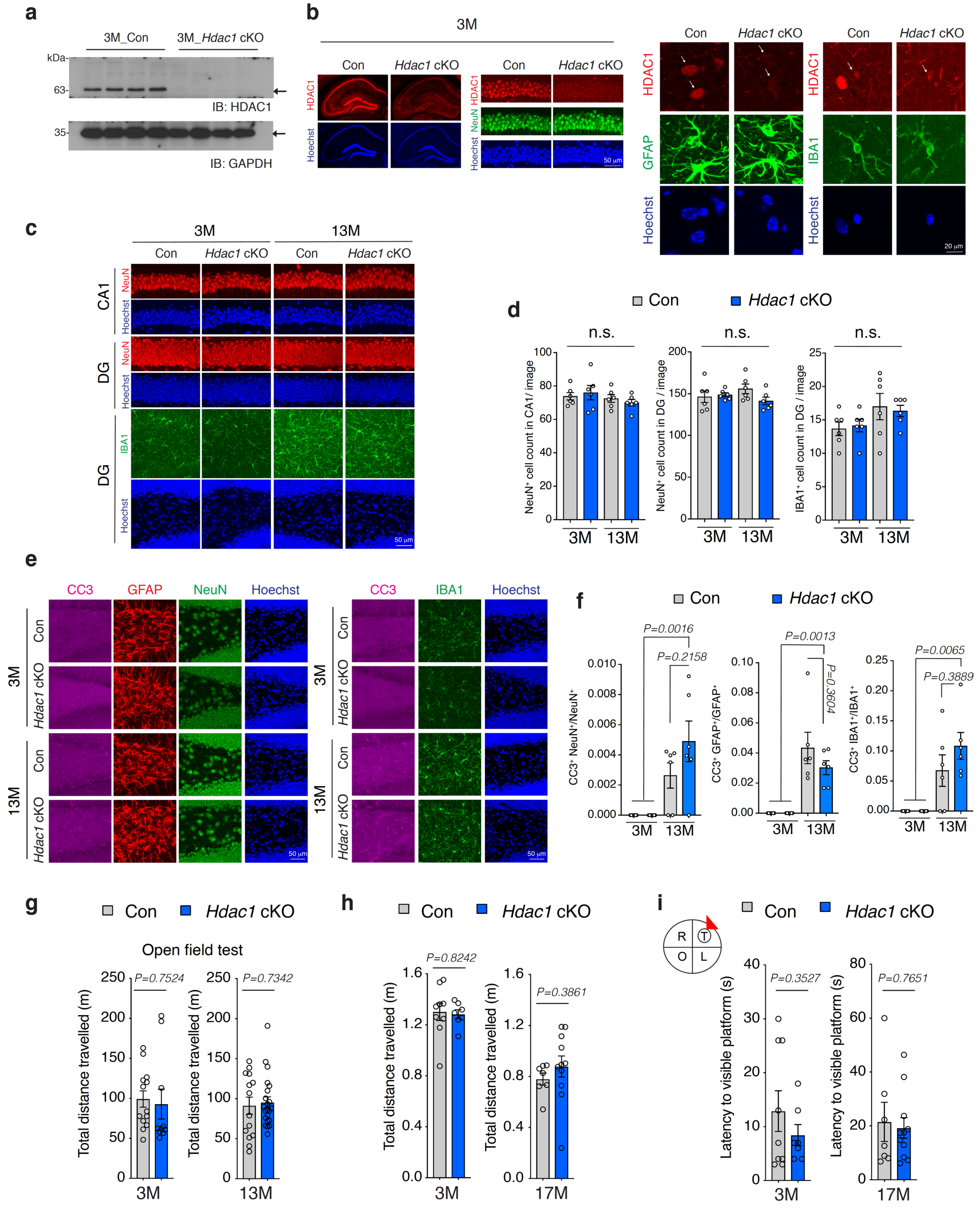
a
3M_Con
3M_Hdac1 cKO
kDa
63
35
IB: HDAC1
IB: GAPDH
b
3M
Con
Hdac1 cKO
HDAC1
NeuN
Hoechst
GFAP
IBA1
50 μm
20 μm
c
3M
13M
CA1
DG
d
Con
Hdac1 cKO
n.s.
NeuN⁺ cell count in CA1/ image
NeuN⁺ cell count in DG / image
IBA1⁺ cell count in DG / image
e
CC3
f
P=0.0016
P=0.2158
P=0.0013
P=0.3604
P=0.0065
P=0.3889
CC3⁺ NeuN⁺/NeuN⁺
CC3⁺ GFAP⁺/GFAP⁺
CC3⁺ IBA1⁺/IBA1⁺
g
Open field test
P=0.7524
P=0.7342
Total distance travelled (m)
h
P=0.8242
P=0.3861
17M
i
R
T
O
L
P=0.3527
P=0.7651
Latency to visible platform (s)

Supplementary Figure 1. Characterization of *Hdac1* cKO mice.

a Analysis of HDAC1 and GAPDH protein levels in the hippocampal lysates from 3M control and *Hdac1* cKO mice. **b** Hippocampal sections from 3M control and *Hdac1* cKO mice stained for HDAC1 (red), cell-type specific markers (NeuN, GFAP, and IBA1; in green) and nuclei Hoechst (blue). White arrows indicate $GFAP^+$ cells (left panels) or $IBA1^+$ cells (right panels) (**a**, **b**) At least two independent experiments. **c** Representative images of hippocampal sections from control and *Hdac1* cKO mice at the age of 3M and 13M stained for NeuN (red), IBA1 (green), and nuclei Hoechst (blue). **d** Quantification plots showing the number of $NeuN^+$ cells and $IBA1^+$ cells in each image. (**c**, **d**) Three animals (two sections/mouse) were analyzed. **e** Representative images of hippocampal sections from control and *Hdac1* cKO mice at the age of 3M and 13M stained for cleaved caspase-3 (CC3, magenta), GFAP (red), NeuN (green), IBA1 (green), and nuclei Hoechst (blue). **f** Quantification plots showing the percentage of $CC3^+$ cells in each cell type. (**e**, **f**) Three animals (two sections/mouse) were analyzed. **g** Analysis of total distance travelled during open field test. Animals analyzed were: 13 (3M control), 10 (3M *Hdac1* cKO), 14 (13M control), 22 (13M *Hdac1* cKO). **h**, **i** Quantification plots showing the total distance travelled during the probe trial and the escape latency to visible platform in the MWM task. (**h**, **i**) Animals analyzed were: 9 (3M control), 7 (3M *Hdac1* cKO), 7 (17M control), 11 (17M *Hdac1* cKO). All values are shown as mean ± SEM. Statistical analysis: (**d**, **f**) One-way ANOVA with Tukey's *post hoc* test, not significant; (**g**-**i**) Two-tailed Student's *t*-test. Source data are provided as a Source Data file. See also Source Data file for statistical information of Fig. S1d.

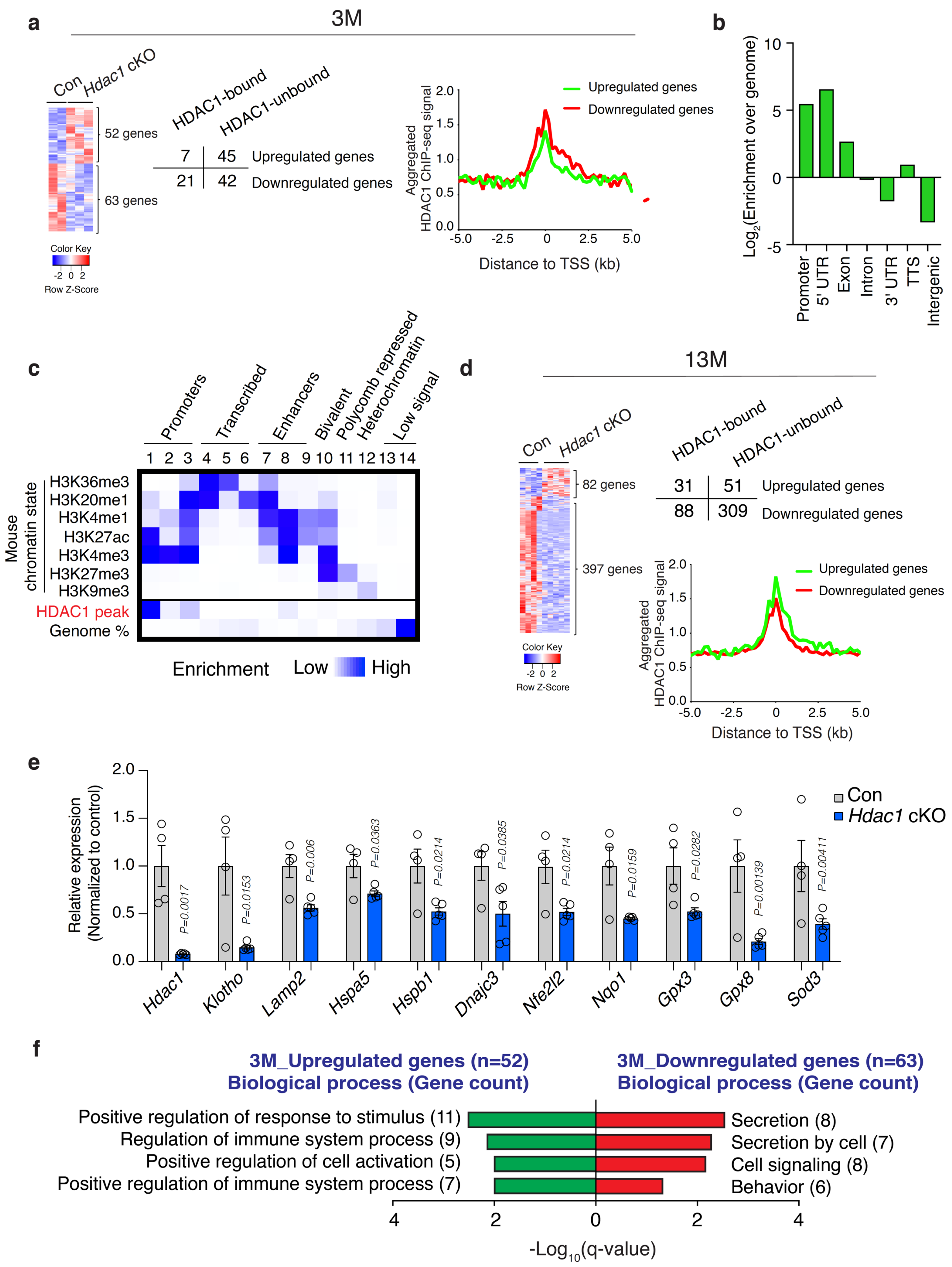
a
3M
Con
Hdac1 cKO
52 genes
63 genes
Color Key
Row Z-Score
HDAC1-bound
HDAC1-unbound
7
45
Upregulated genes
21
42
Downregulated genes
Aggregated HDAC1 ChIP-seq signal
Upregulated genes
Downregulated genes
Distance to TSS (kb)
b
Log2(Enrichment over genome)
Promoter
5' UTR
Exon
Intron
3' UTR
TTS
Intergenic
c
Promoters
Transcribed
Enhancers
Bivalent
Polycomb repressed
Heterochromatin
Low signal
Mouse chromatin state
H3K36me3
H3K20me1
H3K4me1
H3K27ac
H3K4me3
H3K27me3
H3K9me3
HDAC1 peak
Genome %
Enrichment
Low
High
d
13M
Con
Hdac1 cKO
82 genes
397 genes
HDAC1-bound
HDAC1-unbound
31
51
Upregulated genes
88
309
Downregulated genes
Aggregated HDAC1 ChIP-seq signal
Upregulated genes
Downregulated genes
Distance to TSS (kb)
e
Relative expression (Normalized to control)
Con
Hdac1 cKO
P=0.0017
P=0.0153
P=0.006
P=0.0363
P=0.0214
P=0.0385
P=0.0214
P=0.0159
P=0.0282
P=0.00139
P=0.00411
Hdac1
Klotho
Lamp2
Hspa5
Hspb1
Dnajc3
Nfe2l2
Nqo1
Gpx3
Gpx8
Sod3
f
3M_Upregulated genes (n=52)
Biological process (Gene count)
3M_Downregulated genes (n=63)
Biological process (Gene count)
Positive regulation of response to stimulus (11)
Regulation of immune system process (9)
Positive regulation of cell activation (5)
Positive regulation of immune system process (7)
Secretion (8)
Secretion by cell (7)
Cell signaling (8)
Behavior (6)
-Log10(q-value)

Supplementary Figure 2. HDAC1 binds mainly to promoters and validation of gene expression changes identified in RNA-seq.

a A heat map of DEGs identified by RNA-seq of hippocampal tissues from 3M control (n=2) and *Hdac1* cKO (n=3) mice. Aggregated plot of HDAC1 ChIP-seq signal at the transcriptional start site (TSS) of DGEs identified in 3M *Hdac1* cKO mice. Kilo-basepair (kb). **b** Enrichment of HDAC1 binding peaks over genome using HOMER program[1]. UTR, untranslated region; TTS, transcriptional termination site. **c** Enrichment of HDAC1 binding peaks over chromatin states by ChromHMM software[2]. Fourteen types of chromatin states were defined based on our previous ChIP-seq datasets of seven histone marks in wild-type mouse hippocampus[3]. Promoter: active (1), upstream of TSS (2), downstream of TSS (3); Transcribed: transcribed region (4), 3'-transcribed region (5), gene region, not necessarily transcribed (6); Enhancer: within gene region (7), strong (8), weak (9); Bivalent region (10); repressed Polycomb (11); Heterochromatin (12); Low signal: weak gene signal (13), intergenic (14). Dark blue represents a higher enrichment. **d** A heat map of DEGs identified by RNA-seq of hippocampal tissues from 13M control (n=4) and *Hdac1* cKO (n=5) mice. Aggregated plot of HDAC1 ChIP-seq signal at the TSS of DEGs identified in 13M *Hdac1* cKO mice. **e** qPCR analysis to validate gene expression changes identified in RNA-seq. Animals analyzed were: 4 (13M control) and 5 (13M *Hdac1* cKO). **f** Enriched GO terms of DEGs from 3M animals were annotated using MSigDB[4,5]. All values are shown as mean ± SEM. Statistical analysis: (**e**) Two-tailed Student's *t*-test; (**f**) Weighted Kolmogorov-Smirnov-like test followed by multiple hypothesis testing. Source data are provided as a Source Data file.

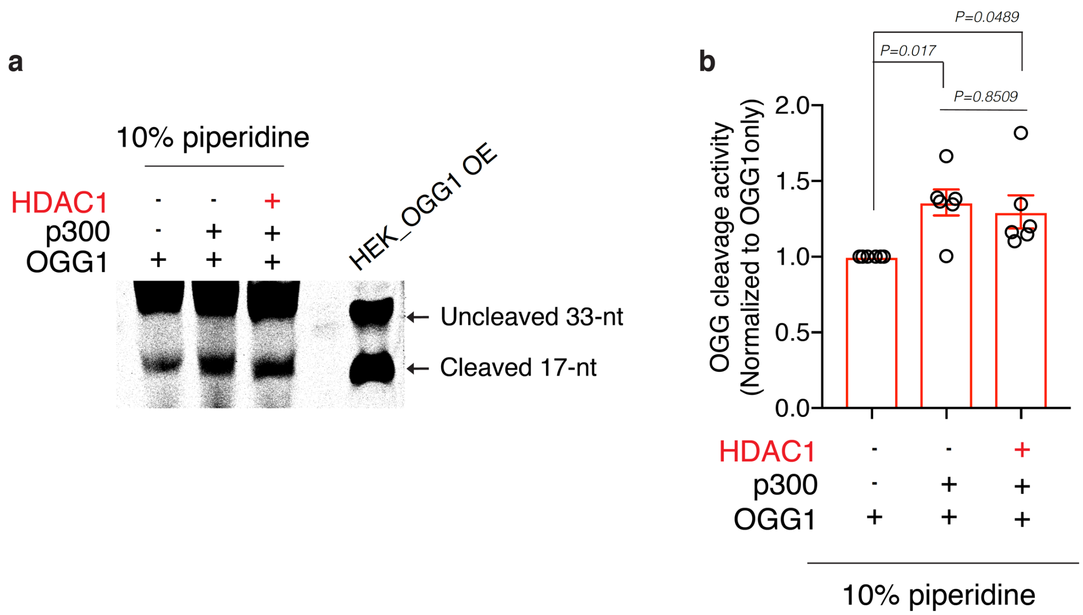

Supplementary Figure 3. Chemical cleavage by piperidine after OGG1 assay to convert all unprocessed AP sites after the reaction.

a, **b** Representative images and quantification of cleaved products after OGG1 activity assay. Samples after OGG1 activity assay were incubated with 10% piperidine to cleave unprocessed AP sites. Six replicates from three independent experiments. All values are shown as mean ± SEM. Statistical analysis: (**b**) One-way ANOVA with Tukey's *post hoc* test. Source data are provided as a Source Data file.

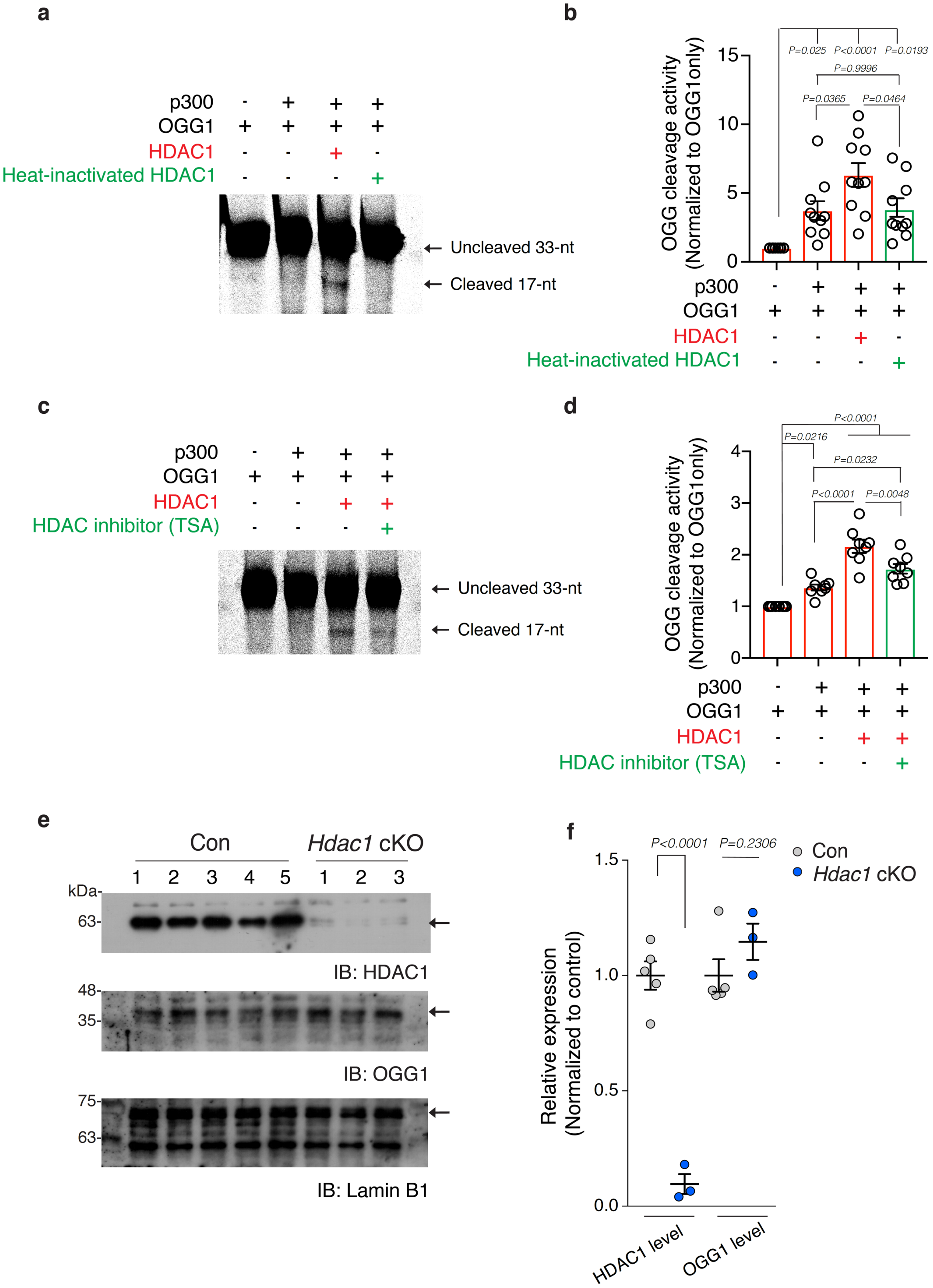
a
p300
OGG1
HDAC1
Heat-inactivated HDAC1
Uncleaved 33-nt
Cleaved 17-nt
b
OGG cleavage activity (Normalized to OGG1only)
P=0.025
P<0.0001
P=0.0193
P=0.9996
P=0.0365
P=0.0464
c
p300
OGG1
HDAC1
HDAC inhibitor (TSA)
Uncleaved 33-nt
Cleaved 17-nt
d
OGG cleavage activity (Normalized to OGG1only)
P<0.0001
P=0.0216
P=0.0232
P<0.0001
P=0.0048
e
Con
Hdac1 cKO
kDa
IB: HDAC1
IB: OGG1
IB: Lamin B1
f
Relative expression (Normalized to control)
P<0.0001
P=0.2306
Con
Hdac1 cKO
HDAC1 level
OGG1 level

Supplementary Figure 4. OGG1 cleavage assay using heat-inactivated HDAC1 or pharmacologically-inhibited HDAC1 and OGG1 protein levels in 13-month-old *Hdac1* cKO mice.

a, **b** Representative images and quantification of OGG1 cleavage activity using heat-inactivated HDAC1. Ten replicates from five independent experiments. **c**, **d** Representative images and quantification of OGG1 cleavage activity in the presence of HDAC inhibitor (100 nM TSA). Eight replicates from four independent experiments. **e**, **f** Western blot analysis of protein levels for HDAC1, OGG1, and Lamin B1 in the hippocampal nuclear extracts from 13M control (n=5) and *Hdac1* cKO (n=3) mice. All values are shown as mean ± SEM. Statistical analysis: (**b**, **d**) One-way ANOVA with Tukey's *post hoc* test; (**f**) Two-tailed Student's *t*-test. Source data are provided as a Source Data file.

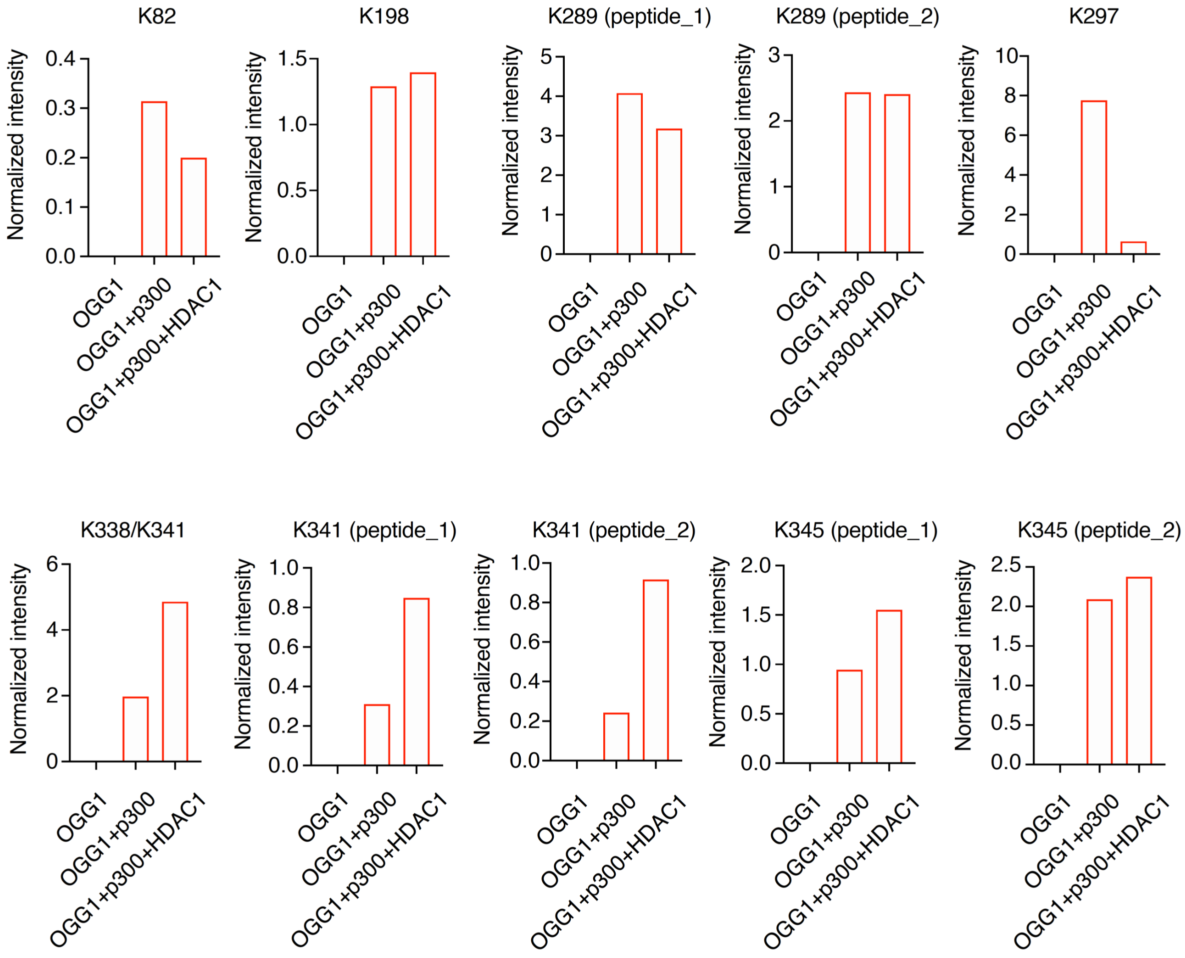

Supplementary Figure 5. Acetylation of OGG1 lysine affected by the presence of p300 and HDAC1.

Shown on the y-axis is acetylation intensity as determined by label-free quantification for each lysine acetylation site using protein abundance-normalized peptide precursor ion intensity. Recombinant OGG1 was treated alone (1st lane), with p300 (2nd lane) or p300 followed by HDAC1 (3rd lane). Source data are provided as a Source Data file.

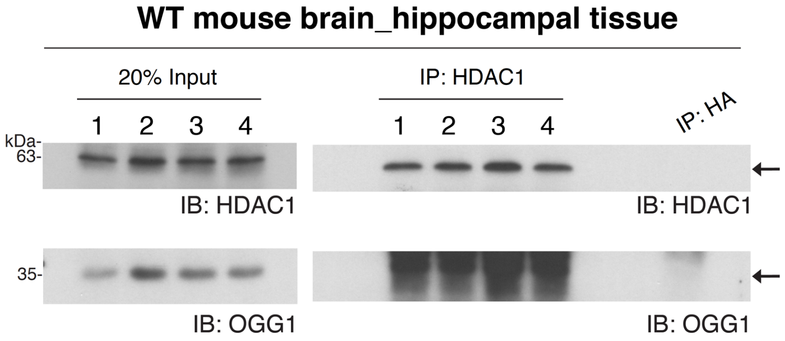

Supplementary Figure 6. Co-immunoprecipitation of HDAC1 with OGG1 in hippocampal nuclear extracts from 2-month-old wild-type animals.

Co-immunoprecipitation of OGG1 with HDAC1 in hippocampal nuclear extracts from 2-month-old wild-type mice (n=4).

a

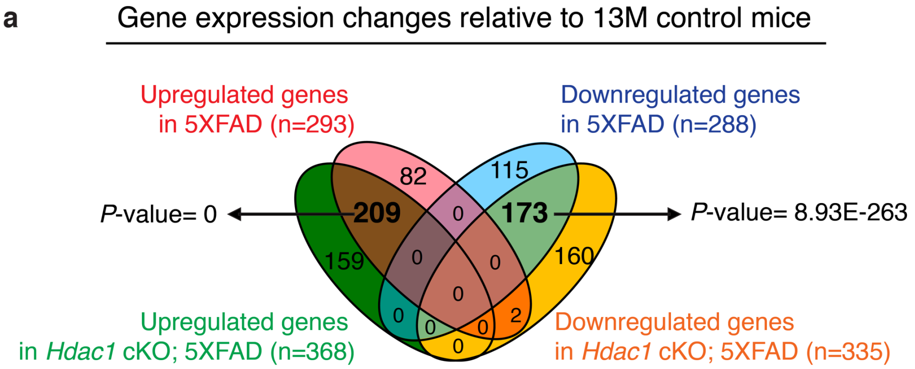

b

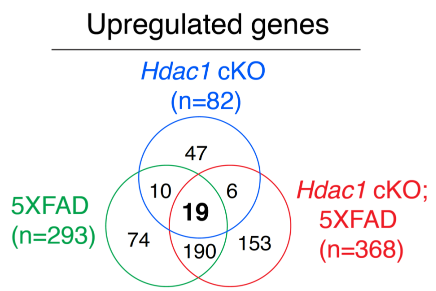

c

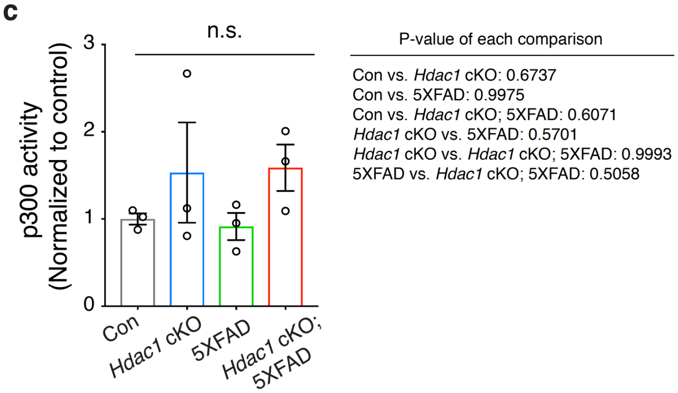

d

5XFAD and *Hdac1* cKO; 5XFAD mice

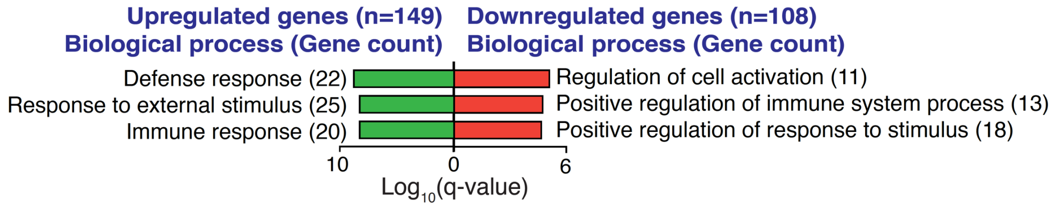

e

5XFAD and *Hdac1* cKO; 5XFAD mice

Upregulated genes (n=149)

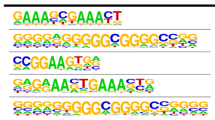

Motif	Name	P-value
	IRF9_MOUSE.H10MO.C	1E-4
	SP1_MOUSE.H10MO.A	1E-4
	GABP1_MOUSE.H10MO.C	1E-4
	IRF8_MOUSE.H10MO.D	1E-3
	SP3_MOUSE.H10MO.B	1E-3

Downregulated genes (n=108)

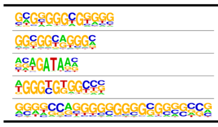

Motif	Name	P-value
	WT1_MOUSE.H10MO.D	1E-2
	EGR4_MOUSE.H10MO.D	1E-2
	GATA4_MOUSE.H10MO.B	1E-2
	KLF4_MOUSE.H10MO.C	1E-2
	SP2_MOUSE.H10MO.D	1E-2

± 500bp from TSS

Supplementary Figure 7. Characterization of differentially expressed genes in 5XFAD and *Hdac1* cKO; 5XFAD mice.

a A Venn diagram showing the overlap in DEGs identified in 13M 5XFAD and *Hdac1* cKO; 5XFAD mice compared to age-matched controls. **b** A Venn diagram showing the overlap in upregulated genes between 13M *Hdac1* cKO, 5XFAD, and *Hdac1* cKO; 5XFAD. **c** Hippocampal tissues from 13M control, *Hdac1* cKO, 5XFAD, and *Hdac1* cKO; 5XFAD mice were assayed for p300 activity. Three animals were analyzed in each group. **d** Enriched GO terms of DEGs identified by MSigDB[4,5] (13M 5XFAD and *Hdac1* cKO; 5XFAD mice). **e** Known motifs identified by HOMER program[1] within ± 500 bp from TSS of DEGs (13M 5XFAD and *Hdac1* cKO; 5XFAD mice). Statistical analysis: (**a**) Two-tailed Fisher's exact test; (**c**) One-way ANOVA with Tukey's *post hoc* test, n.s., not significant; (**d**) Weighted Kolmogorov-Smirnov-like test followed by multiple hypothesis testing; (**e**) Cumulative binomial distributions. Source data are provided as a Source Data file.

a

Group		Cell type	Gene overlaps (# DEGs/# marker genes)	Background overlaps (# marker genes/# total genes)	adj. *P*-value
DEGs in 13M *Hdac1* cKO	UP (n=82)	neuron	0/74	107/21152	N/A
		astrocyte	0/74	41/21152	N/A
		microglia	2/74	356/21152	0.3546
	DN (n=397)	neuron	1/375	107/21152	0.8532
		astrocyte	3/375	41/21152	0.1076
		microglia	10/375	356/21152	0.1549
DEGs in 13M 5XFAD	UP (n=293)	neuron	0/279	107/21152	N/A
		astrocyte	1/279	41/21152	0.4201
		microglia	77/279	356/21152	7.324E-72
	DN (n=288)	neuron	0/270	107/21152	N/A
		astrocyte	3/270	41/21152	0.03068
		microglia	3/270	356/21152	0.8355

b

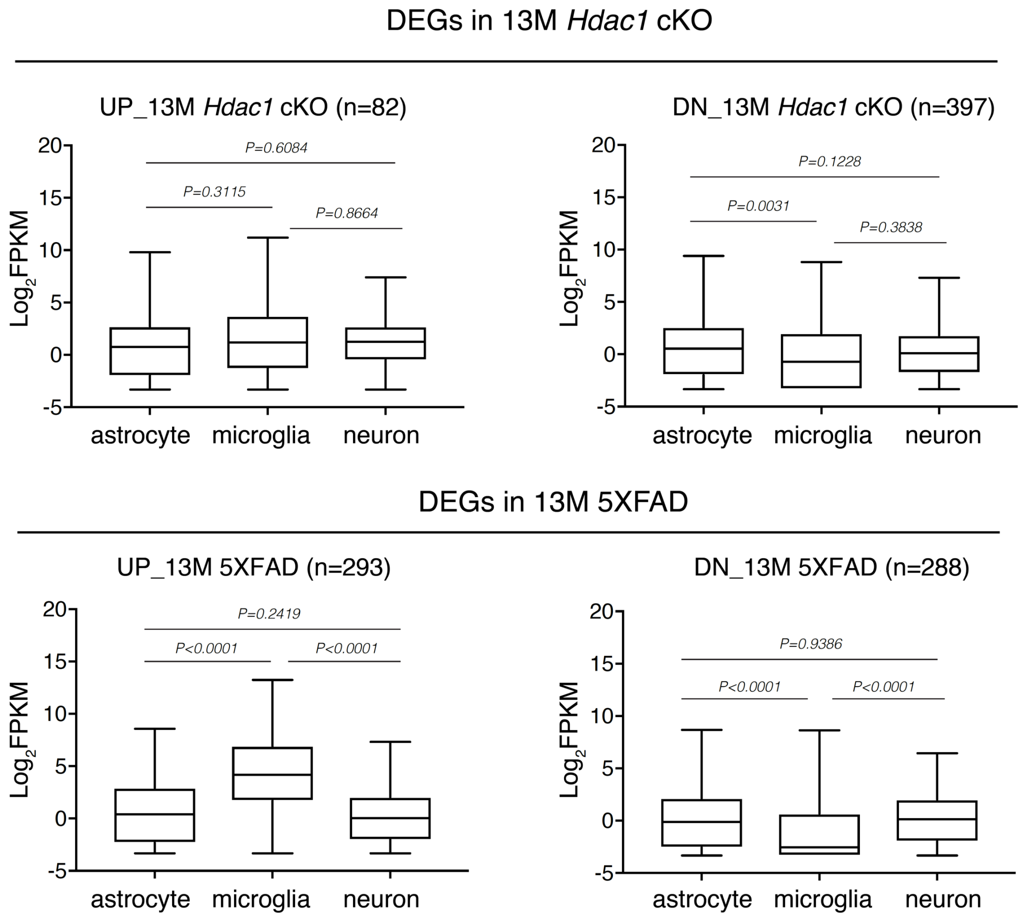

Supplementary Figure 8. Cell-type enrichment analysis of DEGs for 13M *Hdac1* cKO and 5XFAD mice.

a Marker genes for neurons, astrocytes, and microglia were identified using a cell-type specific mouse RNA-seq dataset[6] (https://www.brainrnaseq.org, Brain RNA-seq dataset). Gene overlaps correspond to numbers of DEGs in either *Hdac1* cKO or 5XFAD that were categorized as cell-type marker genes (see also the Method section). **b** Box plots showing the FPKM values of DEGs in each cell-type identified from a cell-type specific mouse RNA-seq dataset[6]. Box-and-whisker plots indicate median, first and third quartile, min and max values. Statistical analysis: (**a**) Two-tailed Fisher's exact test; (**b**) One-way ANOVA followed by Tukey's *post hoc* test. Source data are provided as a Source Data file.

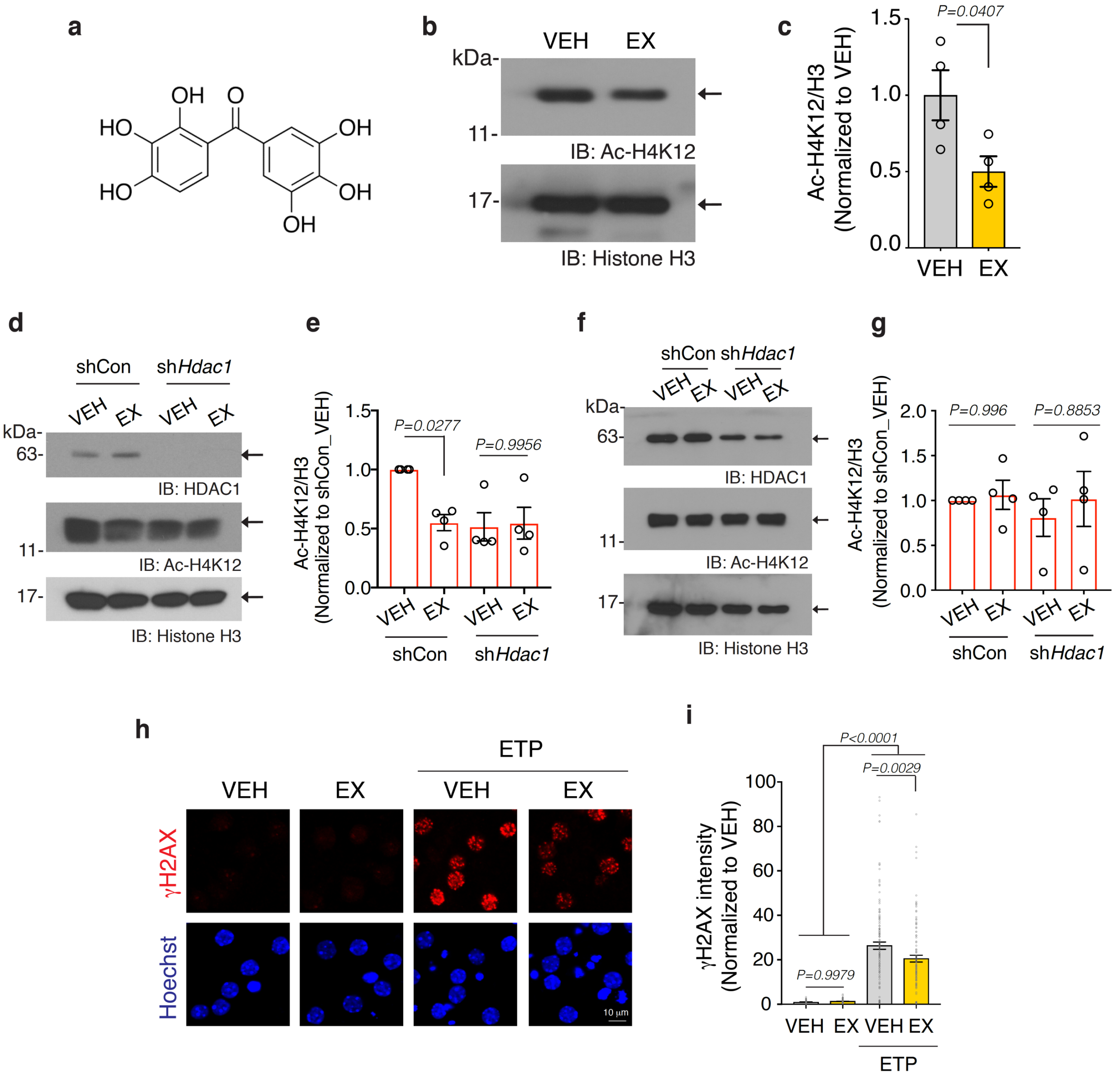

Supplementary Figure 9. Exifone reduces histone acetylation and protects against etoposide-induced DNA damage in cultured neurons.

a Chemical structure of HDAC1 activator, exifone. **b**, **c** Western blot analysis of Ac-H4K12 and Histone H3 levels in the cell lysates of wild-type (WT) cultured neurons (14 DIV) treated with 10 μM exifone (EX) or vehicle (VEH) overnight. (**b**, **c**) Four replicates from

four independent experiments. **d**, **e** Western blot analysis of Ac-H4K12 and Histone H3 levels in the cell lysates of cultured neurons (14 DIV) expressing shCon or sh*Hdac1* that were treated with 10 μM EX or VEH overnight. (**d**, **e**) Four replicates from two independent experiments. **f**, **g** Western blot analysis of Ac-H4K12 and Histone H3 levels in the cell lysates of cultured astrocytes expressing shCon or sh*Hdac1* that were treated with 10 μM EX or VEH overnight. (**f**, **g**) Four replicates from two independent experiments. **h** Representative images of γH2AX immunolabeling in WT cultured neurons (14 DIV) that were preincubated with 10 μM EX or VEH overnight, and then treated with 5 μM etoposide (ETP) for 1 h. **i**, Quantification plot showing the intensity of γH2AX signals. Number of nuclei analyzed were: 97 (VEH), 120 (EX), 137 (VEH+ETP), 112 (EX+ETP). Pyknotic nuclei were filtered out based on nuclei size. All values are shown as mean ± SEM. Statistical analysis: (**c**) Two-tailed Student's *t*-test; (**e**, **g**, **i**) One-way ANOVA with Tukey's *post hoc* test. Source data are provided as a Source Data file.

a

Brain concentration (ng/g)

	Time (h)	
**Animal ID**	**0.5**	**1**
1	42.15	
2	65.37	
3	89.13	
4		35.28
5		50.91
6		42.33
**Mean (ng/g)**	**66.55**	**42.84**

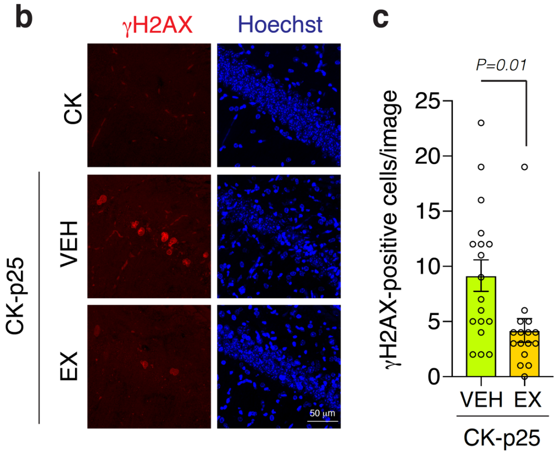

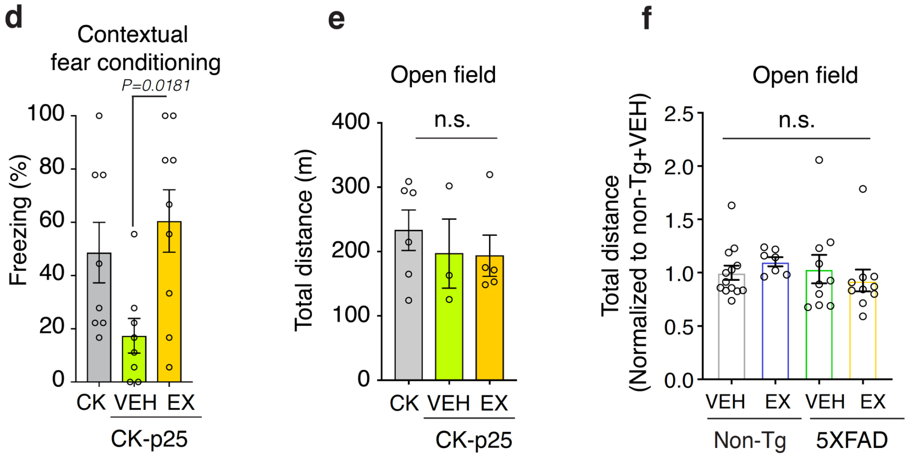

Supplementary Figure 10. Exifone reduces DNA damage and improves cognition in mouse models of neurodegeneration.

a Concentrations of exifone after a single intraperitoneal injection in wild-type mouse brain were measured by liquid chromatography–mass spectrometry (LC/MS/MS). Three animals in each time point were analyzed. **b**, **c** Representative images and quantification of γH2AX immunolabeling in CK, EX-, and VEH-treated CK-p25 mice. CK-p25 mice were induced for 6 weeks, and they received either exifone (50 mg/kg body weight/day) or vehicle for 3 weeks by intraperitoneal injection from week 3 to 6 during the induction

period. (**b**, **c**) Animals analyzed were: 9 (CK-p25+VEH), 8 (CK-p25+EX), and two sections per mouse. Two independent experiments. Hippocampal region. **d** Analysis of freezing behavior during contextual fear conditioning test. CK-p25 mice were induced for 6 weeks, and they received either exifone (50 mg/kg body weight/day) or vehicle for 3 weeks by intraperitoneal injection from week 3 to 6 during the induction period. Animals analyzed were: 8 (CK), 8 (CK-p25+VEH), 9 (CK-p25+EX). **e** Analysis of total distance travelled during open field test. Animals analyzed were: 6 (CK), 3 (CK-p25+VEH), 5 (CK-p25+EX). **f** Analysis of total distance travelled during open field test. Eight-month-old non-Tg control and 5XFAD mice treated with VEH or EX for 2 weeks. Animals analyzed were: 13 (non-Tg+VEH), 7 (non-Tg+EX), 10 (5XFAD+VEH), 10 (5XFAD+EX). All values are shown as mean ± SEM. Statistical analysis: (**c**) Two-tailed Student's *t*-test; (**d**-**f**) One-way ANOVA with Tukey's *post hoc* test, n.s., not significant. Source data are provided as a Source Data file. See also Source Data file for statistical information of Fig. S10d-f.

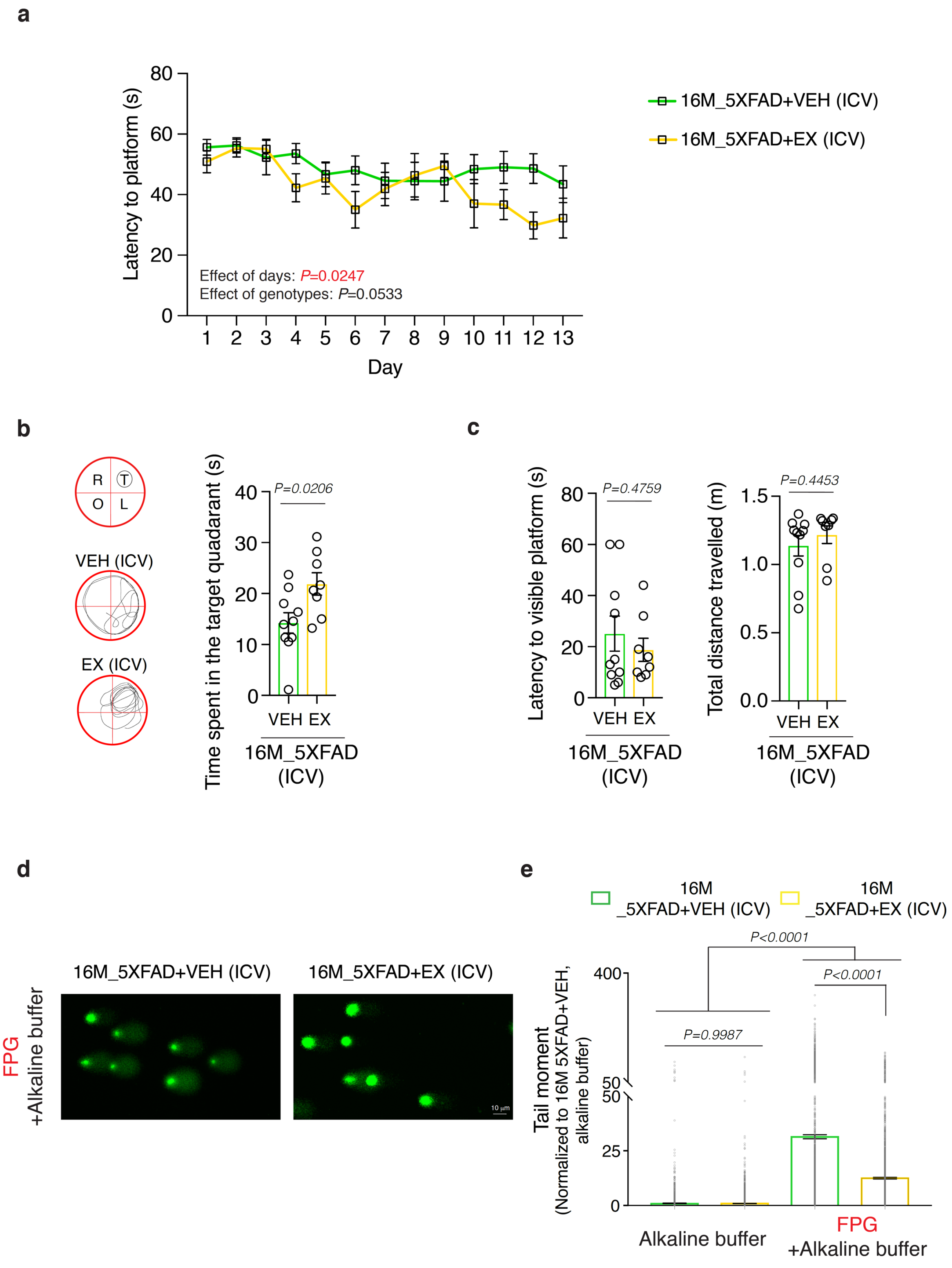
a
Latency to platform (s)
80
60
40
20
0
Effect of days: P=0.0247
Effect of genotypes: P=0.0533
1 2 3 4 5 6 7 8 9 10 11 12 13
Day
16M_5XFAD+VEH (ICV)
16M_5XFAD+EX (ICV)
b
R T O L
VEH (ICV)
EX (ICV)
Time spent in the target quadarant (s)
P=0.0206
VEH EX
16M_5XFAD (ICV)
c
Latency to visible platform (s)
P=0.4759
Total distance travelled (m)
P=0.4453
VEH EX
16M_5XFAD (ICV)
d
16M_5XFAD+VEH (ICV)
16M_5XFAD+EX (ICV)
FPG +Alkaline buffer
10 μm
e
16M _5XFAD+VEH (ICV)
16M _5XFAD+EX (ICV)
P<0.0001
P<0.0001
P=0.9987
Tail moment (Normalized to 16M 5XFAD+VEH, alkaline buffer)
400
50
25
0
Alkaline buffer
FPG +Alkaline buffer

Supplementary Figure 11. Intraventricular delivery of exifone improves memory function and ameliorates hippocampal 8-oxoG lesions in 16-month-old 5XFAD mice. **a**, **b** 16-month-old 5XFAD mice were treated with exifone (EX, 100 ng/day) or vehicle control (VEH) by intracerebroventricular (ICV) injection for 2 weeks and subsequently assessed for spatial learning and memory using the MWM task. Shown are the latency to hidden platform during training and time spent in the target quadrant during the probe trial. Images showing the location of each quadrant and swim trace of mouse during the probe trial. T=target, O=opposite, R=right, L=left. **c** Quantification plots showing the total distance travelled during the probe trial and the escape latency to visible platform in the MWM task. (**a**-**c**) Animals analyzed were: 10 (16M_5XFAD+VEH_ICV), 8 (16M_5XFAD+EX_ICV). **d**, **e** Representative images and quantification of the comet assay using hippocampal homogenates from 16M 5XFAD animals that were treated with EX (100 ng/day) or VEH by ICV injection for 4 weeks. (**d, e**) Nuclei analyzed (three mice/group) were: alkaline, 2,861 (16M_5XFAD+VEH_ICV), 3,849 (16M_5XFAD+EX_ICV); FPG+alkaline, 2,717 (16M_5XFAD+VEH_ICV), 2,597 (16M_5XFAD+EX_ICV). All values are shown as mean ± SEM. Statistical analysis: (**a**) Two-way repeated measures ANOVA with Bonferroni's *post hoc* test for acquisition phase; (**b**, **c**) Two-tailed Student's *t*-test; (**e**) One-way ANOVA with Tukey's *post hoc* test. Source data are provided as a Source Data file.

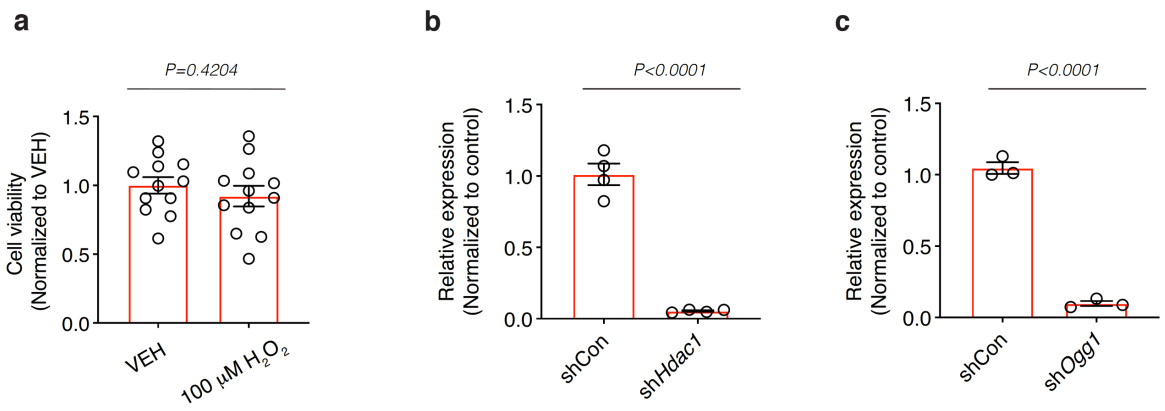

Supplementary Figure 12. Cell viability of cultured neurons after mild oxidative insult and validation of knockdown efficiency in cultured neurons.

a Cell viability was assessed in wild-type cultured neurons (14 DIV) treated with vehicle or 100 μM H_2O_2 overnight. Twelve replicates from two independent experiments. **b**, **c** qPCR analysis to assess knockdown efficiency in cultured neuron (14 DIV) expressing sh*Hdac1* or sh*Ogg1*. (**b**, **c**) At least three replicates from three independent experiments. All values are shown as mean ± SEM. Statistical analysis: (**a**-**c**) Two-tailed Student's *t*-test. Source data are provided as a Source Data file.

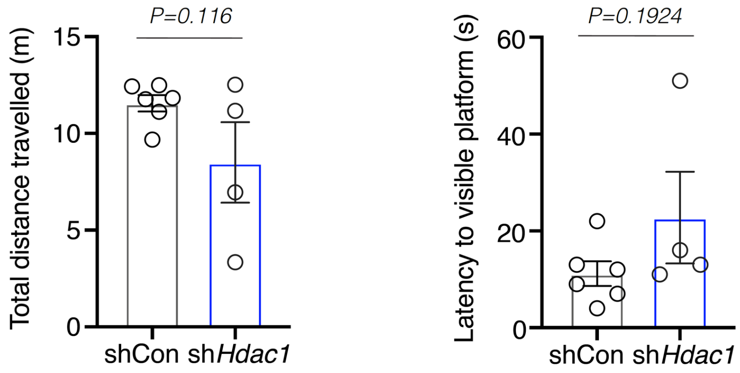

Supplementary Figure 13. Neuronal HDAC1 knockdown has no effects on locomotor activity and vision in aged wild-type animals.

Quantification plots showing the total distance travelled during the probe trial and the escape latency to visible platform in the MWM task. Animals analyzed were: 6 (AAV-shCon-mCherry), 4 (AAV-sh*Hdac1*-mCherry). Statistical analysis: Two-tailed Student's *t*-test. Source data are provided as a Source Data file.

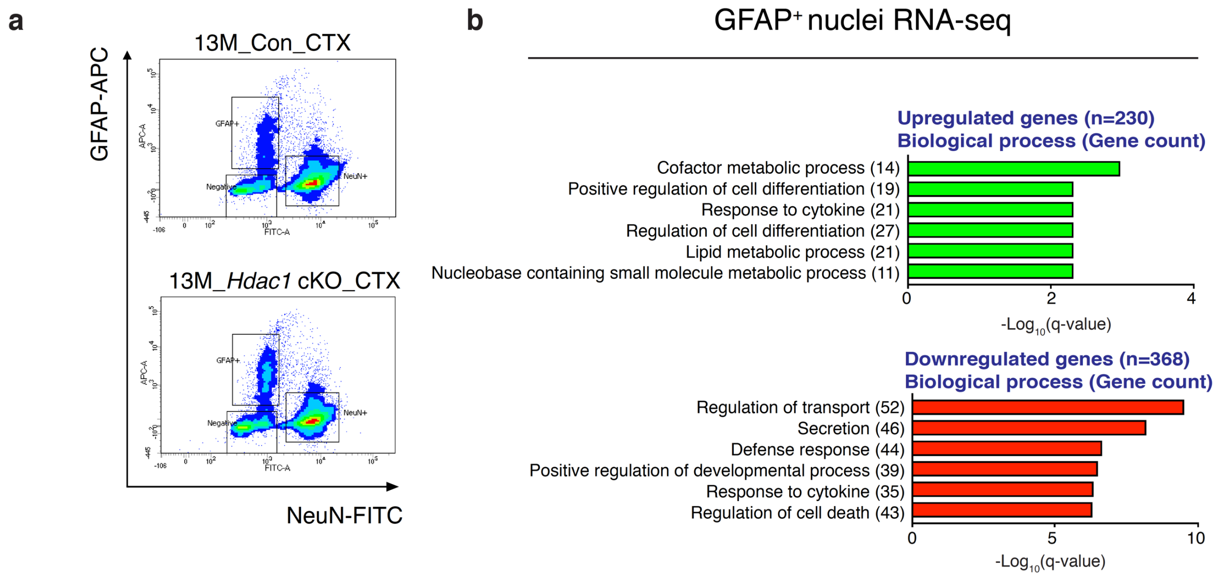

Supplementary Figure 14. Purification and RNA-seq analysis of GFAP⁺ nuclei from aged *Hdac1* cKO and control animals.

a FACS-gating of GFAP⁺ nuclei from cortical tissue of 13-month-old *Hdac1* cKO and control animals. Four animals in each group, and at least 40,000 GFAP⁺ nuclei were isolated from each animal. Cortical tissue was used to obtain enough material for downstream RNA-seq analysis. **b** Enriched GO terms of DEGs were annotated using MSigDB[4,5]. Statistical analysis: (**b**) Weighted Kolmogorov-Smirnov-like test followed by multiple hypothesis testing. Source data are provided as a Source Data file.

Primer	Sequence
Hdac1_F	GACGGCATTGACGACGAATC
Hdac1_R	TGAAGCAACCTAACCGGTCC
Gapdh_F	CATGGCCTTCCGTGTTCCTA
Gapdh_R	CCTGCTTCACCACCTTCTTGAT
Klotho_F	ACTTCGGTGGTCAGGTCAAG
Klotho_R	GGCGGAAAGAGGTGTTGTAG
Lamp2_F	CAACCTGACTCCTGTCGTTC
Lamp2_R	CACAGTGGTGGGAGTTTGG
Hspa5_F	TTGCCATTCAAGGTGGTTG
Hspa5_R	CCAAATACGCCTCAGCAGTC
Hspb1_F	ACTGGCAAGCACGAAGAAAG
Hspb1_R	CTCGAAAGTAACCGGAATGG
Dnajc3_F	ACGCCTTATCACAGTTTC
Dnajc3_R	GTGACCTCTCTGTAGTCTTG
Nfe2l2_F	GCAACTCCAGAAGGAACAGG
Nfe2l2_R	AGGCATCTTGTTTGGGAATG
Nqo1_F	CGAACACAAGAAGCTGGAAG
Nqo1_R	CGAGCACTCTCTCAAACCAG
Gpx3_F	CTGCCTTCTGTCCCTGCTC
Gpx3_R	ATGGTGAGGGCTCCATACTC
Gpx8_F	AAGGATGCCAAAGGAAGGAC
Gpx8_R	GCTCCGATTCTCCAAACTG
Sod3_F	CTGCTCGCTCACATAACAGC
Sod3_R	TGAGGTTCTCTGCACCTGTC
Atp11c_F	TTGTTGGGCGAATCAGTATC
Atp11c_R	AGCCATTTTGGTTTCCATCC
Kcnk10_F	GCACTGAAGGAGGCAAAATC
Kcnk10_R	TGAAGAGAATCGTGGAGATGAC
Slc31a1_F	GAAGAACTCAAGATAGCC
Slc31a1_R	GATGACTACCTGGATGATG
Ogg1_F	ATCATGGCTTCCCAAACCTC
Ogg1_R	GATGCAGTCAGCCACCTTG

Supplementary Table 1.

Primer sequences used for qPCR.

Primer	Sequence
Prkcd_F	TGGGCTCCATTGTGTGTG
Prkcd_R	TTAGCCGTCTCTGGCTCTTG
Gpx3_F	AAGCTGTGTCCTGGGTGTTG
Gpx3_R	CCCCTTTTTCCTCCTCTCC
Slc4a5_F	AAGTGACCAGTTCCTGCTGTC
Slc4a5_R	CTGGATTTGCTGTTTGTACCC
Slc18a2_F	CACACCCTACAGCCTTCATTC
Slc18a2_R	GTTGCTTTCTTCTATGCTCAGG
Cdc25b_F	GCACCGTCCCTTACTGATG
Cdc25b_R	GGAACAGGGTTTGGAGGTG
Sertad1_F	CTACCAGAGCTGTCCCTTCG
Sertad1_R	CACGACTCCAGACACTCGAC
Stk40_F	ACCCAACTTCAGCACTTTCC
Stk40_R	CACCGACTTATGCTCTCCTTG

Supplementary Table 2.

Primer sequences used for ChIP-qPCR.

shRNA	Sequence
shCon	CTTCGAAATGTCCGTTCGGTT
shHdac1	GCCAGTCATGTCCAAAGTAAT
shOgg1	AGTGCTGACCTTCGCCAACCAAGCCTGTT

Supplementary Table 3.

The sequences for shRNAs.

Supplementary References

1. Heinz, S. *et al.* Simple Combinations of Lineage-Determining Transcription Factors Prime cis-Regulatory Elements Required for Macrophage and B Cell Identities. *Mol. Cell* (2010) doi:10.1016/j.molcel.2010.05.004.
2. Ernst, J. & Kellis, M. Discovery and Characterization of Chromatin States for Systematic Annotation of the Human Genome. in *Lecture Notes in Computer Science (including subseries Lecture Notes in Artificial Intelligence and Lecture Notes in Bioinformatics)* (2011). doi:10.1007/978-3-642-20036-6_6.
3. Gjoneska, E. *et al.* Conserved epigenomic signals in mice and humans reveal immune basis of Alzheimer's disease. *Nature* (2015) doi:10.1038/nature14252.
4. Subramanian, A. *et al.* Gene set enrichment analysis: A knowledge-based approach for interpreting genome-wide expression profiles. *Proc. Natl. Acad. Sci.* (2005) doi:10.1073/pnas.0506580102.
5. Mootha, V. K. *et al.* PGC-1α-responsive genes involved in oxidative phosphorylation are coordinately downregulated in human diabetes. *Nat. Genet.* (2003) doi:10.1038/ng1180.
6. Zhang, Y. *et al.* An RNA-sequencing transcriptome and splicing database of glia, neurons, and vascular cells of the cerebral cortex. *J. Neurosci.* (2014) doi:10.1523/JNEUROSCI.1860-14.2014.
