# Supplementary material for: HDAC1 modulates OGG1-initiated oxidative DNA damage repair in the aging brain and Alzheimer’s disease
Source: Nat Commun. 2020 May 18;11:2484. doi: 10.1038/s41467-020-16361-y (PMC7235043; doi:10.1038/s41467-020-16361-y)
Supplement: Supplementary file 2 — Reporting Summary [file 41467_2020_16361_MOESM2_ESM.pdf]

nature research

Corresponding author(s): Li-Huei Tsai

Last updated by author(s): April 8, 2020

Reporting Summary

Nature Research wishes to improve the reproducibility of the work that we publish. This form provides structure for consistency and transparency in reporting. For further information on Nature Research policies, see Authors & Referees and the Editorial Policy Checklist.

Statistics

For all statistical analyses, confirm that the following items are present in the figure legend, table legend, main text, or Methods section.

n/a	Confirmed	
☐	☒	The exact sample size (*n*) for each experimental group/condition, given as a discrete number and unit of measurement
☐	☒	A statement on whether measurements were taken from distinct samples or whether the same sample was measured repeatedly
☐	☒	The statistical test(s) used AND whether they are one- or two-sided<br>*Only common tests should be described solely by name; describe more complex techniques in the Methods section.*
☐	☒	A description of all covariates tested
☒	☐	A description of any assumptions or corrections, such as tests of normality and adjustment for multiple comparisons
☐	☒	A full description of the statistical parameters including central tendency (e.g. means) or other basic estimates (e.g. regression coefficient) AND variation (e.g. standard deviation) or associated estimates of uncertainty (e.g. confidence intervals)
☒	☐	For null hypothesis testing, the test statistic (e.g. *F*, *t*, *r*) with confidence intervals, effect sizes, degrees of freedom and *P* value noted<br>*Give P values as exact values whenever suitable.*
☒	☐	For Bayesian analysis, information on the choice of priors and Markov chain Monte Carlo settings
☒	☐	For hierarchical and complex designs, identification of the appropriate level for tests and full reporting of outcomes
☒	☐	Estimates of effect sizes (e.g. Cohen's *d*, Pearson's *r*), indicating how they were calculated

Our web collection on statistics for biologists contains articles on many of the points above.

Software and code

Policy information about availability of computer code

Data collection	Imaging data were acquired using Zeiss Zen Blue and Zen Black 2012 and Odyssey. Behavior data were acquired using VersaMax software and EthoVision XT. Electrophysiology data were acquired using PULSE software from HEKA.
Data analysis	Analysis of imaging and western blot data was performed using ImageJ software (version 1.51m9). Astrocyte cell volume was analyzed using Imaris image analysis software (version 7.0). For RNA-seq, the collapsed raw fastq reads were aligned by Tophat2, and further processed by Cufflinks 2.0.0 with UCSC mm9 reference gene annotation to determine transcript abundances. Gene ontology (GO) analysis of DEGs were performed using Gene Set Enrichment Analysis (GSEA) for GO biological process (MSigDB, Broad Institute). Transcription factor binding motif analysis of DEGs were conducted using HOMER software. For ChIP-seq, Sequencing reads were aligned to the mouse genome assembly (mm9) using BWA aligner (samse option). Duplicate reads were removed by samtools. Bed alignment files were generated using bedtools. Peak calling was conducted using MACS2 peak-finding algorithm. Annotated genes associated with HDAC1 binding was identified by GREAT software utilizing default settings. MACS2 program was used to generate fold-enrichment of ChIP-seq signals against input across the genome (bedgraph format). The output bedgraph files were used by HOMER program to generate aggregated ChIP-seq signals around gene TSS (5 kb upstream and downstream). Chromatin states were defined by available ChIP-seq datasets, and enrichment of HDAC1 binding over chromatin states was assessed using ChromHMM software. Graphs were generated in GraphPad Prism 7. Statistical analysis was carried out using GraphPad Prism 7. Electrophysiology data were analyzed using pClamp10 (Axon Instruments). Cytometry data were analyzed using BD FACSDiva 8.0 and FlowJo V10 (Tree Star, Inc.). Comet images were analyzed using default settings in OpenComet software, a free plugin for ImageJ (http://www.cometbio.org/index.html).

For manuscripts utilizing custom algorithms or software that are central to the research but not yet described in published literature, software must be made available to editors/reviewers. We strongly encourage code deposition in a community repository (e.g. GitHub). See the Nature Research guidelines for submitting code & software for further information.

Data

Policy information about availability of data

All manuscripts must include a data availability statement. This statement should provide the following information, where applicable:

- Accession codes, unique identifiers, or web links for publicly available datasets
- A list of figures that have associated raw data
- A description of any restrictions on data availability

All data are available from the corresponding author upon reasonable request. The source data are provided as a Source Data file. Raw image files will be deposited in publicly available databases at the time of manuscript publication. Data of RNA-seq and HDAC1 ChIP-seq were deposited in the GEO Data Bank under ID code GSE115437 (https://www.ncbi.nlm.nih.gov/geo/query/acc.cgi?acc=GSE115437) and GSE147407 (https://www.ncbi.nlm.nih.gov/geo/query/acc.cgi?acc=GSE147407).

Field-specific reporting

Please select the one below that is the best fit for your research. If you are not sure, read the appropriate sections before making your selection.

☒ Life sciences ☐ Behavioural & social sciences ☐ Ecological, evolutionary & environmental sciences

For a reference copy of the document with all sections, see nature.com/documents/nr-reporting-summary-flat.pdf

Life sciences study design

All studies must disclose on these points even when the disclosure is negative.

Sample size	The data were assumed to distribute normally, but this was not formally tested. No statistical method was used to determine sample sizes, but sample sizes in this study were similar to previous publications (Kim et al., 2008; Dobbin et al., 2013), except for comet assay (Suberbielle et al., 2013). For the comet assays, at least three animals were used in each group and tile scan images using 5x objective were acquired to obtain all the comets on the slides.<br><br>Kim, D. et al. Deregulation of HDAC1 by p25/Cdk5 in Neurotoxicity. Neuron (2008) doi:10.1016/j.neuron.2008.10.015.<br>Dobbin, M. M. et al. SIRT1 collaborates with ATM and HDAC1 to maintain genomic stability in neurons. Nat. Neurosci. (2013) doi:10.1038/nn.3460.<br>Suberbielle, E. et al. Physiologic brain activity causes DNA double-strand breaks in neurons, with exacerbation by amyloid-β. Nat. Neurosci. (2013) doi:10.1038/nn.3356.
Data exclusions	No data were excluded from the analyses.
Replication	Most of the experiments were carried out at least twice independently. For animal experiments involving only one cohort, at least three animals and multiple sections were used in each group. The OGG1 assay for aged mice after exifone treatment was performed once due to limited availability of aged animals, but four animals were used for each group in this experiment.
Randomization	Animal/samples (mice) were assigned randomly to the various experimental groups, and mice were randomly selected for behavioral experiments.
Blinding	Whenever possible, the investigator performing the analysis was blinded to group allocation during data collection and analysis. However, for some experiments involving limited number of aged animals, the experimenter was aware of the conditions being tested. The analysis of GFAP+ cell volume, 8-oxoG intensity in mouse brain and HDAC1 intensity in mCherry+ cells were performed blind to the conditions of the experiment. Importantly, all mice behavioral data were automatically scored.

Reporting for specific materials, systems and methods

We require information from authors about some types of materials, experimental systems and methods used in many studies. Here, indicate whether each material, system or method listed is relevant to your study. If you are not sure if a list item applies to your research, read the appropriate section before selecting a response.

Materials & experimental systems

n/a	Involved in the study	
☐	☒	Antibodies
☒	☐	Eukaryotic cell lines
☒	☐	Palaeontology
☐	☒	Animals and other organisms
☒	☐	Human research participants
☒	☐	Clinical data

Methods

n/a	Involved in the study	
☐	☒	ChIP-seq
☐	☒	Flow cytometry
☒	☐	MRI-based neuroimaging

Antibodies

Antibodies used

For immunostaining:
anti-HDAC1 polyclonal antibody (Thermo, PA1-860, 1:500)
anti-GFAP polyclonal antibody (Abcam, ab53554, 1:500)
anti-NeuN (Rbfox3) polyclonal antibody (Synaptic Systems, 26604, 1:500)
anti-IBA1 polyclonal antibody (Wako Chemicals, 019-19741, 1:500)
anti-rH2AX monoclonal antibody, clone JBW301 (Millipore, 05-636, 1:500)
anti-8-OHdG monoclonal antibody, clone N45.1 (Cosmo Bio, NNS-MOG-020-EX, 1:100)
and anti-cleaved caspase-3 polyclonal antibody (Abcam, ab2302, 1:200)

For western blot:
anti-HDAC1 polyclonal antibody (Abcam, ab7028, 1:2,000)
anti-OGG1 polyclonal antibody (Novus Biologicals, NB100-106, 1:1,000)
anti-Lamin B1 polyclonal antibody (Abcam, ab16048, 1:1,000)
anti-GAPDH monoclonal antibody, clone 14C10 (Cell Signaling Technology, 2118, 1:5,000)
anti-acetyl-Lysine monoclonal antibody, clone 4G12 (Millipore, 05-515, 1:2,000)
anti-acetyl-Histone H4 (Lys12) polyclonal antibody (Millipore, 07-595, 1:2,000)
anti-Histone H3 monoclonal antibody, clone mAbcam 24834 (Abcam, ab24834, 1:10,000)
and anti-p300 monoclonal antibody, clone RW128 (ThermoFisher, MA1-16608, 1:1,000)

For immunoprecipitation:
anti-OGG1/2 monoclonal antibody, clone G-5 (Santa Cruz Biotechnology, sc-376935)
anti-HDAC1 monoclonal antibody, clone 10E2 (Cell Signaling Technology, 5356)
anti-8-OHdG monoclonal antibody, clone N45.1 (Cosmo Bio, NNS-MOG-020-EX)
anti-OGG1 polyclonal antibody (Novus Biologicals, NB100-106)
anti-HDAC1 polyclonal antibody (Abcam, ab7028)
anti-p300 monoclonal antibody, clone RW128 (ThermoFisher, MA1-16608)
and anti-Normal Mouse IgG polyclonal antibody (Millipore, 12-371)

For nuclei sorting:
anti-GFAP monoclonal antibody, clone GA5, Alexa Fluor® 647 Conjugated (Cell Signaling Technology, 3657)
and anti-NeuN monoclonal antibody, clone A60, Alexa Fluor® 488 Conjugated (Millipore, MAB377X)

Secondary antibody for western blot:
anti-Rabbit IgG HRP Linked Whole antibody (GE Healthcare, NXA931)
and anti-Rabbit IgG HRP Linked Whole antibody (GE Healthcare, NA934)

Secondary antibody for immunostaining:
Donkey anti-Mouse IgG (H+L) Highly Cross-Adsorbed Secondary Antibody, Alexa Fluor 488 (Thermo Fisher Scientific, A-21202)
Goat anti-Mouse IgG (H+L) Highly Cross-Adsorbed Secondary Antibody, Alexa Fluor 594
(Thermo Fisher Scientific, A-11032)
Donkey anti-Rabbit IgG (H+L) Highly Cross-Adsorbed Secondary Antibody, Alexa Fluor 488 (Thermo Fisher Scientific, A-21206)
Goat anti-Rabbit IgG (H+L) Highly Cross-Adsorbed Secondary Antibody, Alexa Fluor 594
(Thermo Fisher Scientific, A-11037)
Goat anti-Guinea Pig IgG (H+L) Highly Cross-Adsorbed Secondary Antibody, Alexa Fluor 647 (Thermo Fisher Scientific, A-21450)
and Donkey anti-Goat IgG (H+L) Cross-Adsorbed Secondary Antibody, Alexa Fluor 488
(Thermo Fisher Scientific, A-11055)

Validation

For immunostaining:
anti-HDAC1 polyclonal antibody (Thermo, PA1-860)
Species Reactivity: Dog, Hamster, Human, Mouse, Rat
Validated applications: ChIP, ICC, IF, IP, WB

anti-GFAP polyclonal antibody (Abcam, ab53554, 1:500)
Species Reactivity: Mouse, Rat, Human, Zebrafish
Validated applications: WB, IHC, ICC, IF

anti-NeuN (Rbfox3) polyclonal antibody (Synaptic Systems, 26604, 1:500)
Species Reactivity: Mouse, Rat, Human
Validated applications: IHC, ICC

anti-IBA1 polyclonal antibody (Wako Chemicals, 019-19741, 1:500)
Species Reactivity: Mouse, Rat, Human
Validated applications: IHC, IF, ICC

anti-rH2AX monoclonal antibody, clone JBW301 (Millipore, 05-636, 1:500)
Species Reactivity: Mouse
Validated applications: IHC, IF, ICC, WB, ChIP

anti-8-OHdG monoclonal antibody, clone N45.1 (Cosmo Bio, NNS-MOG-020-EX, 1:100)
Species Reactivity: Mouse

Validated applications: IHC

and anti-cleaved caspase-3 polyclonal antibody (Abcam, ab2302, 1:200)
Species Reactivity: Rabbit, Human, Quail
Validated applications: IHC, WB

For western blot:
anti-HDAC1 polyclonal antibody (Abcam, ab7028, 1:2,000)
Species Reactivity: Mouse, Rat, Human
Validated applications: IHC, IF, ICC, WB, ChIP, IP

anti-OGG1 polyclonal antibody (Novus Biologicals, NB100-106, 1:1,000)
Species Reactivity: Mouse, Rat, Human, Rabbit, Primate
Validated applications: IHC, IF, ICC, WB, ChIP, IP, ELISA, Flow, IB

anti-Lamin B1 polyclonal antibody (Abcam, ab16048, 1:1,000)
Species Reactivity: Mouse, Rat, Human, Pig, Xenopus laevis, Indian muntjac
Validated applications: IHC, IF, ICC, WB

anti-GAPDH monoclonal antibody, clone 14C10 (Cell Signaling Technology, 2118, 1:5,000)
Species Reactivity: Mouse, Rat, Human, Pig, Monkey, Bovine
Validated applications: IHC, IF, ICC, WB, Flow

anti-acetyl-Lysine monoclonal antibody, clone 4G12 (Millipore, 05-515, 1:2,000)
Species Reactivity: Mouse, Rat, Human
Validated applications: IP, WB

anti-acetyl-Histone H4 (Lys12) polyclonal antibody (Millipore, 07-595, 1:2,000)
Species Reactivity: Yeast, Human
Validated applications: WB, ChIP

anti-Histone H3 monoclonal antibody, clone mAbcam 24834 (Abcam, ab24834, 1:10,000)
Species Reactivity: Mouse, Human, Rat
Validated applications: WB, ChIP

and anti-p300 monoclonal antibody, clone RW128 (ThermoFisher, MA1-16608, 1:1,000)
Species Reactivity: Human, Mustelid, Mouse, Non-human primate, Rat
Validated applications: WB, ICC, IF, IP

For immunoprecipitation:
anti-OGG1/2 monoclonal antibody, clone G-5 (Santa Cruz Biotechnology, sc-376935)
Species Reactivity: Human, Mouse, Rat
Validated applications: WB, ICC, IF, ELISA

anti-HDAC1 monoclonal antibody, clone 10E2 (Cell Signaling Technology, 5356)
Species Reactivity: Human, Mouse, Rat, Monkey
Validated applications: WB, IP

anti-8-OHdG monoclonal antibody, clone N45.1 (Cosmo Bio, NNS-MOG-020-EX)
Species Reactivity: Mouse
Validated applications: IHC

anti-OGG1 polyclonal antibody (Novus Biologicals, NB100-106)
Species Reactivity: Mouse, Rat, Human, Rabbit, Primate
Validated applications: IHC, IF, ICC, WB, ChIP, IP, ELISA, Flow, IB

anti-HDAC1 polyclonal antibody (Abcam, ab7028)
Species Reactivity: Mouse, Rat, Human
Validated applications: IHC, IF, ICC, WB, ChIP, IP

anti-p300 monoclonal antibody, clone RW128 (ThermoFisher, MA1-16608)
Species Reactivity: Human, Mustelid, Mouse, Non-human primate, Rat
Validated applications: WB, ICC, IF, IP

and anti-Normal Mouse IgG polyclonal antibody (Millipore, 12-371)
Validated applications: WB, IP

For nuclei sorting:
anti-GFAP monoclonal antibody, clone GA5, Alexa Fluor® 647 Conjugate (Cell Signaling Technology, 3657)
Species Reactivity: Mouse, Rat, Human
Validated applications: IF

and anti-NeuN monoclonal antibody, clone A60, Alexa Fluor® 488 Conjugated (Millipore, MAB377X)
Species Reactivity: Mouse, Rat, Human

Validated applications: IHC

Animals and other organisms

Policy information about studies involving animals; ARRIVE guidelines recommended for reporting animal research

Laboratory animals	Hdac1f/f mice were gifted from E.N. Olson at UTSW. Nestin-Cre transgenic and 5XFAD mice were obtained from Jackson Laboratory (JAX ID# 03771, 34840). Aged C57BL/6J mice were obtained from National Institute on Aging. Two to 3-month-old male mice were used as young group, and 13 to 17-month-old male mice were used as aged group. Mice were housed in groups of 3-5 animals on a standard 12 h light / 12 h dark cycle, temperature (24°C), and humidity (45%) with food and water available ad libitum. All experiments were performed during the light cycle.
Wild animals	The study did not involve wild animals.
Field-collected samples	The study did not involve samples collected from the field.
Ethics oversight	Massachusetts Institute of Technology: Institutional Animal Care and Use Committee (IACUC) & Comm on Animal Care (CAC).

Note that full information on the approval of the study protocol must also be provided in the manuscript.

ChIP-seq

Data deposition

☒ Confirm that both raw and final processed data have been deposited in a public database such as GEO.

☒ Confirm that you have deposited or provided access to graph files (e.g. BED files) for the called peaks.

Data access links<br>*May remain private before publication.*	https://www.ncbi.nlm.nih.gov/geo/query/acc.cgi?acc=GSE115437
Files in database submission	GSM3178267 ChIP-Seq WCE_rep1<br>GSM3178268 ChIP-Seq WCE_rep2<br>GSM3178269 ChIP-Seq WCE_rep3<br>GSM3178270 ChIP-Seq HDAC1_rep1<br>GSM3178271 ChIP-Seq HDAC1_rep2<br>GSM3178272 ChIP-Seq HDAC1_rep3
Genome browser session<br>(e.g. UCSC)	No longer applicable

Methodology

Replicates	Three replicates per group.
Sequencing depth	The mean yield per sample was 15,647,780 40-bp single-end reads, of which 12,979,186 reads were aligned (82.9%).
Antibodies	anti-HDAC1 monoclonal antibody, clone 10E2 (Cell Signaling Technology, 5356)
Peak calling parameters	MACS2 peak-finding algorithm
Data quality	We identified 6,998 peaks (0.05% FDR cut-off), and 798 of them were above 5-fold enrichment.
Software	For ChIP-seq, Sequencing reads were aligned to the mouse genome assembly (mm9) using BWA aligner (samse option). Duplicate reads were removed by samtools. Bed alignment files were generated using bedtools. Peak calling was conducted using MACS2 peak-finding algorithm. Annotated genes associated with HDAC1 binding was identified by GREAT software utilizing default settings. MACS2 program was used to generate fold-enrichment of ChIP-seq signals against input across the genome (bedgraph format). The output bedgraph files were used by HOMER program to generate aggregated ChIP-seq signals around gene TSS (5 kb upstream and downstream). Chromatin states were defined by available ChIP-seq datasets, and enrichment of HDAC1 binding over chromatin states was assessed using ChromHMM software.

Flow Cytometry

Plots

Confirm that:

☒ The axis labels state the marker and fluorochrome used (e.g. CD4-FITC).

☒ The axis scales are clearly visible. Include numbers along axes only for bottom left plot of group (a 'group' is an analysis of identical markers).

☒ All plots are contour plots with outliers or pseudocolor plots.

☒ A numerical value for number of cells or percentage (with statistics) is provided.

Methodology

Sample preparation	Frozen cortical tissue was homogenized using a dounce tissue grinder (Wheaton) in 1 mL of cold NF1 buffer (10 mM Tris-HCl pH8.0, 5 mM MgCl2, 0.1 M sucrose, 1 mM EDTA pH8.0, 0.5% Triton X-100, proteinase inhibitor cocktail), and the suspension was centrifuged at 1,600 x g at 4°C for 7 min. Pellets were incubated with 10 mL of NF1 buffer at 4 °C for 30 min with shaking to release nuclei. Released nuclei were then pelleted down at 1,600 x g for 7 min and incubated with Alexa flour APC conjugated GFAP antibody (Cell Signaling Technology, 3657, 1:200) and Alexa flour 488 conjugated NeuN (Millipore, MAB377X, 1:200) in cold in cold PBS with 1% BSA and proteinase inhibitor cocktail on a tube rotator at 4 °C for 3 h. Pelleted nuclei were then resuspended in 0.5 mL PBS and were run through a 0.45 um cell strainer prior to FACS sorting.
Instrument	BD FACSAria Flow Cytometer
Software	Cytometry data were analyzed using BD FACSDiva 8.0 and FlowJo V10 (Tree Star, Inc.).
Cell population abundance	The GFAP+ nuclei are about 7 to 10% from the cortical homogenates.
Gating strategy	FSC-A/SSC-A gates of the starting cell population were used to discriminate between viable cells and cell debris. Singlet and doublet cells were discriminated using FSC-A/ Pacific Blue-A gating.

☐ Tick this box to confirm that a figure exemplifying the gating strategy is provided in the Supplementary Information.
